# A study protocol for determining the role of Sympathetic activity in Post-injury outcomes: Impact on sleep and caRdiovascular health InvesTigation (SPIRIT)

**DOI:** 10.1371/journal.pone.0321035

**Published:** 2025-07-17

**Authors:** Lauren E. Walker, Sarah M. Jurick, Victoria Thomas, Thomas Arnold, Katerina Elisman, Sachi Kalra, Autumn Mains, Stephanie Rioux, Michael Galarneau, Cameron McCabe, Jason M. Lavender, Jacob Collen, Mark C. Haigney, Soroosh Solhjoo, Ian J. Stewart

**Affiliations:** 1 Military Cardiovascular Outcomes Research (MiCOR), Bethesda, Maryland, United States of America; 2 Metis Foundation, San Antonio, Texas, United States of America; 3 Naval Health Research Center, San Diego, California, United States of America; 4 Leidos, San Diego, California, United States of America; 5 Department of Biostatistics, Brown University, Providence, Rhode Island, United States of America; 6 Department of Medicine, Uniformed Services University of the Health Sciences, Bethesda, Maryland, United States of America; 7 F. Edward Hébert School of Medicine, Bethesda, Maryland, United States of America; University of Health Sciences, Beyhekim Training and Research Hospital, TÜRKIYE

## Abstract

**Introduction:**

A growing body of research indicates that combat-injured service members are at heightened risk of a wide range of adverse physical and mental health outcomes. However, data on long-term health outcomes of United States (US) combat-injured service members remain limited, particularly in relation to cardiovascular outcomes and potential underlying mechanisms. The goal of the “*determining the role of sympathetic activity in post-injury outcomes: impact on sleep and cardiovascular health investigation (SPIRIT)”* study is to better understand the relationship between combat injury severity and cardiovascular function, sleep disorders, and mental health concerns among post-9/11 US combat casualties.

**Methods:**

Here, we describe a remotely conducted, nationwide, cross-sectional study of 100 minimally injured (Injury Severity Score [ISS] ≤ 3) and 100 severely injured (ISS ≥ 14) post-9/11 US combat casualties. Injury and demographic data will be collected from both retrospective sources and the participants themselves. Participants will complete well-validated surveys electronically to assess mental health symptoms and sleep problems. Three wearable devices will be mailed to participants: (1) an ambulatory electrocardiogram (ECG) monitor for seven days of wear, (2) a Home Sleep Test (HST) device to be worn for one night, and (3) a 24-hour ambulatory blood pressure monitor to be worn for one day. Lastly, participants will visit a local laboratory to provide blood and urine samples for analysis. This protocol is registered on Clinicaltrials.gov under #NCT05971433.

**Results and conclusions:**

This study will be among the first to remotely evaluate long-term physical and mental health outcomes in US combat casualties. This study aims to collect robust at-home ECG, sleep, and blood pressure data and examine the associations of combat injury severity with sleep disorders, mental health, cardiovascular risk, and sympathetic nervous system activation. Findings will help inform future research, such as interventional or subsequent longitudinal observational investigations of combat-injured veterans.

## Introduction

More than 53,000 United States (US) service members have been injured in combat since the onset of post-9/11 conflicts in Iraq and Afghanistan [1]. Emerging evidence from retrospective studies demonstrates that combat casualties are at an increased risk for a wide variety of adverse outcomes, including hypertension (HTN) [2–5], cardiovascular disease (CVD), sleep disorders [6,7], and mental health concerns such as posttraumatic stress disorder (PTSD) [8,9]. Here, we describe the methods for a new, remotely conducted, cross-sectional protocol examining long-term physical and mental health outcomes in post-9/11 combat-injured service members.

Early research on the association of combat injury severity and health outcomes focused on cardiovascular health, examining the relationship between combat injury and subsequent diagnoses of HTN and CVD in a cohort of 3,846 combat-injured service members who required care in an intensive care unit (ICU) [2]. The injury severity score (ISS), a validated, anatomy-based scoring system ranging from 1–75 [10], was used to quantify the severity of traumatic injury. Each five-point increase in ISS was associated with a 6% increase in the rate of HTN and a 13% increase in the rate of CVD. A more recent investigation built on this work by comparing combat-injured participants randomly selected from the Department of Defense (DoD) trauma registry to a control group of service members who were deployed but not injured (total study N = 17,454) [3]. Injury severity was categorized as mild-to-moderate (ISS < 25) or severe (ISS ≥ 25). In a series of multivariable models that adjusted for potential confounders, rates of HTN were higher for the mild-to-moderate (hazard ratio [HR] 1.14, 95% confidence interval [CI] 1.05–1.24; P = 0.002) and severe (HR 2.78, 95% CI 2.18–3.55; P < 0.001) injury groups compared to controls. The rates of CVD were also higher in the mild-to-moderate (HR 1.65, 95% CI 1.11–2.37; P = 0.013) and severe (HR 4.87, 95% CI 2.11–11.25; P < 0.001) injury groups compared to controls after adjustment. With a median follow-up time of 8.4 years, these data suggest that combat injury represents a long-term risk factor for both HTN and CVD, even several years after the initial injury.

Further research into the cohort described above found increased rates of obstructive sleep apnea (OSA) among service members with deployment-related combat injuries compared to those who deployed and did not have combat injuries. The relationship appeared to be driven by comorbid traumatic brain injury (TBI) and mental health sequelae of traumatic injury [6]. OSA is an established cause of HTN, likely driven by increased sympathetic tone, and the loss of the healthy nocturnal cardiovascular “dipping phenomena.” Traumatic injury also predicted higher rates of insomnia compared to non-injured service members, even after accounting for mental health diagnoses [7]. What was most concerning about these findings was the increased frequency of sleep disorders among injured compared to non-injured service members for a decade post-injury, emphasizing the importance of longer-term screening in this population.

The preponderance of studies examining long-term health outcomes of combat-injured service members has been fully retrospective, utilizing administrative and medical record data. Since combat casualties are seen by healthcare providers more frequently than their uninjured counterparts, ascertainment bias may be an issue. Secondly, administrative records do not usually provide the data necessary for examining potential mechanisms underlying observed associations. Therefore, a crucial next step is a cross-sectional study to validate the associations observed in prior retrospective studies. The geographical dispersion of this patient population presents a unique challenge, complicating both recruitment and data collection; thus, investigators in the present study previously conducted a pilot study to assess the feasibility of remote data collection in combat casualties [11]. The pilot study recruited a subset of participants who were already enrolled in the Wounded Warrior Recovery Project (WWRP), an ongoing, web-based investigation of injured service members conducted by the Naval Health Research Center (NHRC), in which participants provide self-reported outcomes [12,13]. The pilot study successfully enrolled and obtained laboratory data from 119 participants from 38 states and the territory of Puerto Rico over roughly ten months, confirming the feasibility of a remote recruitment and data collection approach in this population.

The precise mechanisms by which combat injury may increase the risk of developing HTN and CVD are unclear. Several potential pathways have been proposed, however, including mental health concerns, sleep disorders, and inflammation [2,3]. Furthermore, accumulating evidence suggests that sympathetic nervous system (SNS) hyperactivity may underlie HTN and have bidirectional influences with sleep disorders [3,14–17]. In the present work, we describe the protocol design for a cross-sectional study, *determining the role of Sympathetic activity in Post-injury outcomes: Impact on sleep and caRdiovascular health InvesTigation (SPIRIT)*, which aims to examine HTN, CVD, SNS activation, mental health concerns, and sleep disorders in minimally injured (ISS ≤ 3) and severely injured (ISS ≥ 14) combat casualties. We hypothesize that more severe combat injury will be associated with a higher degree of sleep disorders, mental health symptoms and associated behavioral health problems, SNS hyperactivity, arrhythmia burden, and increased inflammation.

## Methods

The SPIRIT study is a cross-sectional investigation of severely (ISS ≥ 14, n = 100) and minimally (ISS ≤ 3, n = 100) injured post-9/11 service members who sustained combat injuries in Iraq or Afghanistan. Data on mental health, sleep characteristics, and cardiovascular health will be collected remotely at a single time point. The study protocol was reviewed and approved by the Uniformed Services University of the Health Sciences (USUHS) Institutional Review Board (IRB) with concurrence from the NHRC IRB. The protocol was registered on ClinicalTrials.gov on 02/08/2023 with the identification number NCT05971433. The recruitment period began on 03/04/2024 and will tentatively end on 06/08/2025.

### Recruitment

Service members who were injured in combat will be eligible for this study if they are (1) at least 18 years old, enrolled in WWRP, and previously agreed to be contacted about future research opportunities, and (2) able to complete the informed consent process, receive mailed study devices, and complete laboratory procedures within the US. Subjects that are unable to complete the informed consent process, be mailed study devices, or complete laboratory draws within the US will be excluded. Two groups of combat-injured participants (n = 100 per group) will be recruited. This study has been powered to detect an unadjusted period prevalence difference of 0.15 between the severely injured (ISS ≥ 14; assumed 25% prevalence) and minimally injured (ISS ≤ 3; assumed 10% prevalence) groups, with n = 100 per group, two-sided α = 0.05, and 80% power. The first group will comprise severely injured participants with an ISS ≥ 14. If enrollment targets are not met with this cutoff score, the study team will consider enrolling participants with an ISS ≥ 11 in the severely injured group. The second group will include minimally injured participants, defined as ISS ≤ 3. Cutoff scores were determined by assessing the ISS distribution among eligible WWRP participants, and then estimating the number of SPIRIT study candidates needed in each group to enroll 100 participants per group. Based on previous recruitment rates in the aforementioned pilot study [11], we estimated the expected enrollment rate for the SPIRIT study to be roughly 30%. NHRC staff have shared contact information for potentially eligible WWRP participants with the USUHS Military Cardiovascular Outcomes Research (MiCOR) Program team. Research staff at MiCOR will contact study candidates via phone to assess their willingness to participate, verify eligibility, and schedule a remote study visit to complete the informed consent process. Participants will review and sign the informed consent and Health Insurance Portability and Accountability Act of 1996 (HIPAA) authorization forms using a digital signature tool in REDCap (Research Electronic Data Capture) hosted at USUHS [18,19].

### Study population

Demographic and injury data for the eligible pool of WWRP participants, stratified by injury severity, are presented in Table 1. The two groups are similar in terms of age at injury, race and ethnicity, marital status, education, rank, and military status. The median (interquartile range [IQR]) ISS for the minimally and severely injured groups is 1 (1–2) and 20 (17–27), respectively. Compared to minimally injured WWRP participants, severely injured WWRP participants have a longer interval since injury (16.6 vs 15.6 years), are more likely to be male (98.6% vs 92.9%), and in the US Army (75.5% vs 63.2%). Groups also differed by mechanisms of injury (p <.001). Consistent with more extensive injuries, the severely injured group has higher rates of amputations ranging from 3.3% to 12.3% for upper and lower extremities, respectively, while there were no reported amputations in the minimally injured group. Conversely, the minimally injured group has higher rates of mild TBI (32.2% vs 25.3%).

**Table 1:** Characteristics of WWRP participants eligible for recruitment into the SPIRIT study.

Characteristics	Overall (N = 2519)	ISS ≤ 3 (*n* = 2160)	ISS ≥ 14 (*n* = 359)	*P* Value^b^
Age at injury, years (median, IQR)	25.2 (22.2-31.0)	25.2 (22.2-30.8)	25.4 (22.6-32.1)	0.190
Time since injury, years (median, IQR)	15.9 (12.1-17.8)	15.6 (12.0-17.8)	16.6 (13.2-18.3)	**<0.001**
Current age, years (median, IQR)^a^	40.7 (37.4-46.1)	40.5 (37.3-45.9)	41.7 (38.1-47.9)	**<0.001**
Sex				**<0.001**
Male, N (%)	2360 (93.7)	2006 (92.9)	354 (98.6)	
Female, N (%)	154 (6.1)	149 (6.9)	5 (1.4)	
Missing, N (%)	5 (0.2)	5 (0.2)	0 (0.0)	
Race/ethnicity				0.117
Black/African American, N (%)	189 (7.5)	171 (7.9)	18 (5.0)	
Hispanic/Latino, N (%)	261 (10.4)	230 (10.6)	31 (8.6)	
Non-Hispanic White, N (%)	1900 (75.4)	1615 (74.8)	285 (79.4)	
Other, N (%)	136 (5.4)	119 (5.5)	17 (4.7)	
Missing, N (%)	33 (1.3)	25 (1.2)	8 (2.2)	
Marital status				0.595
Married, N (%)	1835 (72.8)	1575 (72.9)	260 (72.4)	
Divorced, separated, or widowed, N (%)	112 (4.4)	99 (4.6)	13 (3.6)	
Never married, N (%)	567 (22.5)	481 (22.3)	86 (24.0)	
Missing, N (%)	5 (0.2)	5 (0.2)	0 (0.0)	
Education				0.973
High school/equivalent or less, N (%)	2010 (79.8)	1724 (79.8)	286 (79.7)	
Some college, N (%)	88 (3.5)	76 (3.5)	12 (3.3)	
Bachelor’s or higher, N (%)	399 (15.8)	341 (15.8)	58 (16.2)	
Missing, N (%)	22 (0.9)	19 (0.9)	3 (0.8)	
Service branch^c^				**<0.001**
Air Force, N (%)	51 (2.0)	47 (2.2)	4 (1.1)	
Army, N (%)	1637 (65.0)	1366 (63.2)	271 (75.5)	
Marine Corps, N (%)	726 (28.8)	650 (30.1)	76 (21.2)	
Navy, N (%)	100 (4.0)	92 (4.3)	8 (2.2)	
Missing, N (%)	5 (0.2)	5 (0.2)	0 (0.0)	
Rank				0.660
Junior enlisted, N (%)	1033 (41.0)	879 (40.7)	154 (42.9)	
Senior enlisted, N (%)	11799 (46.8)	1013 (46.9)	166 (46.2)	
Officer, N (%)	302 (12.0)	263 (12.2)	39 (10.9)	
Missing, N (%)	5 (0.2)	5 (0.2)	0 (0.0)	
Military status				0.903
Active duty/National Guard, N (%)	653 (25.9)	559 (25.9)	94 (26.2)	
Separated/retired, N (%)	1866 (74.1)	1601 (74.1)	265 (73.8)	
ISS (median, IQR)	2.00 (1.0-3.0)	1.00 (1.0-2.0)	20.00 (17.0-27.0)	**<0.001**
Mechanism of Injury				**<0.001**
Blast, N (%)	1861 (73.9)	1566 (72.5)	295 (82.2)	
Gunshot wound, N (%)	289 (11.5)	242 (11.2)	47 (13.1)	
Other, N (%)	306 (12.1)	292 (13.5)	14 (3.9)	
Missing, N (%)	63 (2.5)	60 (2.8)	3 (0.8)	
Deployment-related TBI^c^				**<0.001**
Moderate/severe, N (%)	3 (0.1)	0 (0.0)	3 (0.8)	
Mild, N (%)	787 (31.2)	696 (32.2)	91 (25.3)	
No TBI history, N (%)	1728 (68.6)	1464 (67.8)	264 (73.5)	
Missing, N (%)	1 (0.0)	0 (0.0)	1 (0.3)	
Amputations^c^				
Upper Extremity, N (%)	12 (0.5)	0 (0)	12 (3.3)	**<0.001**
Unilateral, N (%)	12 (0.5)	0 (0)	12 (3.3)	**<0.001**
Bilateral, N (%)	1 (0)	0 (0)	1 (0.3)	0.143
Lower Extremity, N (%)	44 (1.7)	0 (0)	44 (12.3)	**<0.001**
Unilateral, N (%)	32 (1.3)	0 (0)	32 (8.9)	**<0.001**
Bilateral, N (%)	12 (0.5)	0 (0)	12 (3.3)	**<0.001**
Above Knee, N (%)	19 (0.8)	0 (0)	19 (5.3)	**<0.001**
Below Knee, N (%)	29 (1.2)	0 (0)	29 (8.1)	**<0.001**

WWRP, Wounded Warrior Recovery Project; SPIRIT, Sympathetic activity in Post-injury outcomes: Impact on sleep and caRdiovascular health InvesTigation; ISS, injury severity score; IQR, interquartile range; TBI, traumatic brain injury

a Current age as of Sept 15, 2023

b Mann-Whitney U tests were conducted for continuous variables, and Chi-square tests were conducted for categorical variables. *P* values < 0.001 are bolded to denote significance.

c Fisher’s exact test was used due to count < 5

### Data collection

Data that will be collected for the SPIRIT study are outlined in Table 2. We will obtain sociodemographics (date of birth, sex, race/ethnicity, military rank, marital status, address, income and education, and military deployments), medical history (smoking and drug use, family history of premature CVD, and a review of current and past medications and diagnoses), and some injury information (amputations and TBI history) directly from participants during the initial phone interview. Additional demographics and injury characteristics (age, time since injury, ISS, mechanism of injury, amputations, and TBI history, as available) were obtained from the Expeditionary Medical Encounters Database (EMED) at NHRC, which contains medical records of service members deployed during or after 2001 [20]. Data from EMED was transferred to the USUHS study team along with contact information for potential study candidates from WWRP. Participants who enroll in the SPIRIT study will use a secure link to the USUHS’s REDCap to electronically complete validated mental and behavioral health questionnaires assessing PTSD symptoms, depressive symptoms, dissociative experiences, and sleep characteristics. PTSD symptoms will be assessed by the 20-item PTSD Checklist for DSM-5 (PCL-5) [21], depressive symptoms will be measured by the eight-item Patient Health Questionnaire 8 (PHQ-8) [22], and dissociative experiences will be assessed using the eight-item modified Brief Dissociative Experience Scale (DES-B) – Modified [23]. Participant-reported sleep characteristics will be assessed using the Pittsburgh Sleep Quality Index (PSQI) [24], the Epworth Sleepiness Scale (ESS) [25], the Insomnia Severity Index (ISI) [26], and the Berlin Sleep Questionnaire [27].

**Table 2:** Data collection.

Collection Method	Data description
Participant Interview	Sociodemographics Date of Birth Sex Race/ethnicity Military Rank Marital status Mailing address Socioeconomic status (income and education) Military deployments history Medical history Smoking and drug use Personal/family history of premature cardiovascular disease Medications and past medical history Injury characteristics Amputations history TBI history
NHRC data transfer	Injury characteristics and additional demographics Age Time since injury ISS Mechanism of injury Amputation status TBI history
Patient-reported outcomes (mental health and sleep)	PTSD Checklist for DSM-5 (PCL-5) Brief Dissociative Experience Scale (DES-B) Patient Health Questionnaire 8 (PHQ-8) Pittsburgh Sleep Quality Index (PSQI) Epworth Sleepiness Scale (ESS) Insomnia Severity Index (ISI) Berlin Sleep Questionnaire
Laboratory visit	Blood pressure, height, weight, body mass index, and waist circumference High-sensitivity C-reactive protein Lipid panel Hemoglobin A1C Albumin/creatinine ratio Comprehensive metabolic panel Cystatin C with estimated glomerular filtration rate N-terminal pro b-type natriuretic peptide
Wearable devices	Ambulatory electrocardiogram (ECG) monitor 24-hour ambulatory blood pressure monitor (ABPM) Home sleep test (HST)

TBI, traumatic brain injury; NHRC, Naval Health Research Center; ISS, Injury Severity Score; PTSD, posttraumatic stress disorder

Participants will visit a contracted Clinical Laboratory Improvement Amendments of the 1988-certified laboratory of their choosing to provide blood and urine samples to examine high sensitivity C-reactive protein (hs-CRP), lipid panel, hemoglobin A1C, albumin/creatinine ratio, comprehensive metabolic panel, cystatin C with estimated glomerular filtration rate, and N-terminal pro b-type natriuretic peptide. Participants’ blood pressure, height, weight, and waist circumference will also be measured during the laboratory visit.

Participants will be mailed three wearable devices with instructions for use: (1) an ambulatory electrocardiogram (ECG) monitor to be worn for seven consecutive days; (2) a Home Sleep Test (HST) device to be worn on the second or third study day, and (3) a 24-hour ambulatory blood pressure monitor (ABPM) to be worn on the day following the HST. Participants will subsequently mail all wearable devices back to the respective service providers or the USUHS study team, as appropriate, who will complete the device data extraction. The ambulatory ECG monitor will record one-lead ECGs to evaluate SNS activation. In addition to heart rate and heart rate variability (HRV) measures, QT intervals and their variability (QTV) will be measured. The ambulatory ECG will also determine the incidence and nature of arrhythmias, including premature ventricular contractions (PVC), the incidence of atrial fibrillation, as well as atrial and ventricular tachycardia. The frequency of episodes of atrial fibrillation or atrial flutter lasting longer than five minutes, the PVC density (percentage of total heartbeats that are PVCs), and the frequency of ventricular tachycardia will be captured from these reports. Heart rate, HRV, and QTV measures will be automatically calculated for each five-minute epoch of the ECG recordings. HRV time-domain measures include the standard deviation of beat-to-beat intervals (SDNN) and root mean square of successive differences (RMSSD). HRV frequency domain measures include low-frequency (LF, 0.04–0.15 Hz) and high-frequency (HF, 0.15–0.4 Hz) power components and the LF/HF ratio. QTV measures include log-transformed QT variance (logQTv) and beat-to-beat QT variability index (QTVI).

The presence of comorbid OSA and nocturnal hypoxemia will be evaluated with the HST. The HST device will be worn during sleep on the second study day. Blood pressure will be measured using the 24-hour ABPM, the gold standard for assessing HTN [28]. Mean 24-hour blood pressure and mean blood pressure during sleep have the most robust association with long-term major cardiovascular events among all blood pressure-related measures [29]. The ABPM will be worn during the 24 hours following the HST. The mean blood pressure for the entire 24 hours of wear and the sleep time will be computed. Nocturnal dipping and the drop in blood pressure during sleep will also be assessed.

### Objectives and hypotheses

The primary objectives of this study are two-fold: firstly, we will collect robust ambulatory ECG and blood pressure data at a single time point from cohorts of severely injured (ISS ≥ 14, n = 100) and minimally injured (ISS ≤ 3, n = 100) current and former service members; secondly, we will examine the associations between ECG variables, sleep characteristics, mental health, SNS activity, and physical health outcomes including HTN. We will test the following five hypotheses:

*Hypothesis 1:* Severely injured service members have higher cardiovascular risk as defined by the pooled cohort equation (a tool for estimating the 10-year risk of CVD, it includes age, race, total cholesterol, HDL cholesterol, systolic blood pressure, blood pressure medications, diabetes, smoking [30]) and hsCRP (a validated serologic marker of CVD risk [31,32]) compared to minimally injured service members.

*Hypothesis 2:* Severely injured service members have higher levels of SNS activity and arrhythmia burden (as evaluated by ECG monitor) compared to minimally injured service members.

*Hypothesis 3:* Severely injured service members have higher 24-hour mean blood pressure and less nocturnal dipping (as evaluated by ABPM) compared to minimally injured service members.

*Hypothesis 4:* (1) The prevalence of both insomnia symptoms (as defined by ESS and ISI) and OSA (as defined by HST) is greater among severely injured service members compared to minimally injured service members, and (2) the presence of OSA among injured service members correlates strongly with HTN and other cardiovascular comorbidities.

*Hypothesis 5:* Mental health symptoms (PTSD symptoms, depressive symptoms, and dissociative experiences, as defined by the PCL-5, PHQ-8, and DES-B) will be significantly associated with SNS hyperactivity, sleep disorders, and cardiovascular risk, and this association will differ by injury severity.

### Statistical plan

Summary statistics and visualizations will be produced to describe the variables of interest. Study participants (N=200) will be characterized in terms of demographics and clinical variables using percentages for categorical variables and either means and standard deviations or median and interquartile range for continuous variables. We will assess differences in characteristic distributions by group using independent *t* tests or corresponding nonparametric tests (Wilcoxon Rank Sum) and Chi-squared tests (or Fisher’s Exact test for small sample sizes), as appropriate. Two-tailed tests will be performed using the R Stats Package (version 4.4.1). *P* values will be reported with three decimal digits, and *P* < 0.05 will be considered significant. We will evaluate the study hypotheses using univariable and multivariable linear mixed-effects models (LMM) and logistic regression. LMMs will be used for continuous outcomes (hypotheses 1, 2, and 3 for continuous blood pressure). Logistic regression models will be used for binary outcomes (hypothesis 3 for nocturnal dipping, hypothesis 4). In the case of ordinal outcomes, proportional odds cumulative logit (POCL) will be the statistical test of choice (hypothesis 5). Each outcome will be analyzed individually.

## Discussion

In this cross-sectional study of minimally and severely injured combat casualties living throughout the US, we will collect ambulatory, at-home (ECG, HST, and 24-hour ABPM), laboratory, and survey-based data on cardiovascular function, sleep behavior, mental health, SNS activation, and physical health outcomes. The primary objectives of this study will be to (1) collect robust ambulatory ECG and blood pressure data from the target study population and (2) use these data to examine combat injury severity in relation to the identified mental, behavioral, and physical health outcomes.

Given that many of the more than 53,000 post-9/11 combat casualties are still living with the long-term effects of their injuries, and that future large-scale combat operations could result in many more casualties [33], it is critical to better define and identify targets for intervention to reduce long-term health risks among combat-injured military personnel and veterans.

**Fig 1:**
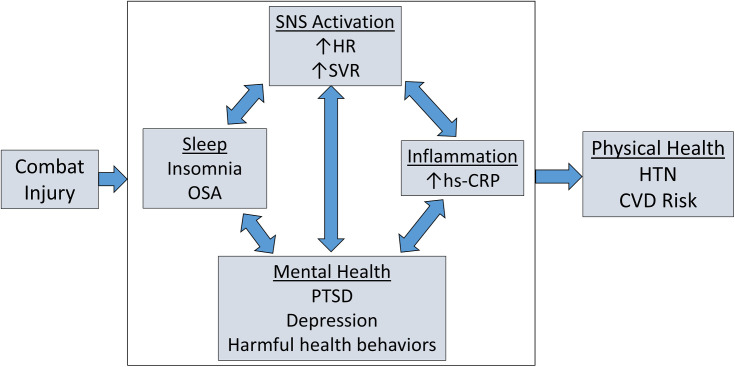
The conceptual framework for the pathways between combat injury and subsequent adverse physical health outcomes. SNS, Sympathetic nervous system; HR, Heart rate; SVR, Systemic vascular resistance; OSA, Obstructive sleep apnea; PTSD, Posttraumatic stress disorder; hs-CRP, High-sensitivity C-reactive protein; HTN, Hypertension; CVD, Cardiovascular disease.

While CVD, HTN, sleep disorders, and mental health concerns are more common in injured service members compared to non-injured service members, it is important to note that these various outcomes do not occur in isolation and that the relationships may be bidirectional. For example, insomnia is associated with subsequent PTSD, depression, and anxiety [8], and mental health conditions increase the risk for subsequent insomnia [7]. Furthermore, PTSD has been associated with both activation of the SNS [34] and inflammation [35] in veterans, while markers of SNS activation are associated with both insomnia [36] and inflammatory markers [37]. Sleep disorders, which are common in military and veteran populations [38,39], are associated with an increased risk of HTN [40], cardiac arrhythmias [41], and sudden cardiac death [42,43]. Poor quality sleep and diagnosed sleep disorders may, therefore, represent a common pathway for both cardiometabolic disease and mental health outcomes among survivors of combat-related injury, as well as a potential target for therapeutic interventions. Given this body of evidence, the mechanisms that link sleep disorders and mental health conditions to CVD and HTN are likely to be complex and interrelated, as shown in our conceptual model (Fig 1). Data collected during the SPIRIT study will be used to test multiple hypotheses related to this conceptual model.

### Related ongoing studies

The Long-term Impact of Military-Relevant Brain Injury Consortium - Chronic Effects of Neurotrauma Consortium (LIMBIC-CENC) Prospective Longitudinal Study (PLS) is perhaps the largest ongoing, prospective investigation of injured US service members, with over 1,700 combat veterans enrolled as of 2021, and a goal of 3,000 participants by 2024 [44]. However, the primary exposure of interest in the LIMBIC-CENC PLS is mild TBI sustained in combat, with more than 80% of participants having a history of at least one mild TBI. Other large, longitudinal studies of US veterans of recent conflicts have either focused on a single service branch and primarily examined mental and behavioral health outcomes [45], or in the case of the WWRP, examined the effects of combat injury using only patient-reported outcomes and retrospective record review (i.e., no objective, laboratory, blood-based or wearable device data) [13].

Outside the US, the Armed Services Trauma and Rehabilitation Outcome (ADVANCE) research initiative is a large-scale longitudinal study of mental and physical health outcomes in male combat-injured service members from the United Kingdom (UK). Using a variety of in-person tests and survey assessments, investigators from ADVANCE are examining the long-term impact of combat injury on outcomes such as musculoskeletal disease, cardiovascular risk and disease, and mental health outcomes in 600 UK service members injured in Afghanistan matched to 600 combat-deployed, non-injured service members [46]. Since the start of enrollment in 2016, findings from the ADVANCE study have already shown associations between combat injury and subsequent long-term health outcomes, including mental health and cardiovascular outcomes [47–52].

Like SPIRIT, ADVANCE collects laboratory data to include blood counts, chemistries, hemoglobin A1C, hsCRP, and lipids, evaluates blood pressure and HRV, and uses validated survey measures to assess sleep. Although ADVANCE and SPIRIT both utilize physical health and self-report measures, SPIRIT will supplement ADVANCE in several ways. Most importantly, ambulatory/at-home devices will be used to collect additional data in SPIRIT, including an ambulatory ECG monitor worn for seven consecutive days (compared to two consecutive five-minute recordings in ADVANCE), a 24-hour ABPM, which is the gold standard for the diagnosis of HTN (compared to central and peripheral blood pressure measurements in ADVANCE), and an HST device (compared to no sleep test in ADVANCE). Using these wearable devices will allow us to obtain, with high granularity, robust measurements of cardiovascular function and sleep disturbances. Moreover, wearable devices (compared to in-person tests in ADVANCE) will enable us to recruit broadly from the overall population of combat-injured service members throughout the US. Without remote recruitment and participation, the SPIRIT study would be limited to either studying only participants living in a particular geographic region or enrolling only participants willing and able to travel to a study site. Lastly, currently, the ADVANCE study only enrolls male UK service members. SPIRIT, however, will enroll both male and female combat-injured service members.

### Limitations

There are several limitations to the SPIRIT study. First, the cross-sectional design will only examine one point in time. Future longitudinal studies are required to better define the risk of adverse long-term outcomes in combat casualties and the prospective nature of associations among different risk factors. Second, the overall number of participants is modest (n = 100 per group), which will limit statistical power, particularly for analyses involving less-common outcomes or low prevalence injuries (e.g., TBI). Third, since the study is observational, the findings will be correlative and cannot be used to infer causation. Lastly, there may be selection bias as subjects who respond to the invitation may differ from those who decline the invitation to participate in the study. On completion of recruitment, subjects who opted to be enrolled in the study will be compared to those who did not to assess for potential selection bias.

### Future research

The long-term goal of this research effort is to identify the most promising targets for future interventional investigations, such as randomized controlled trials (RCT), to improve outcomes in survivors of combat injury. These targets could include aggressive treatment of sleep-disordered breathing and hypoxemia by instituting early continuous positive airway pressure (CPAP), more aggressive treatment of HTN, more aggressive treatment of PTSD, or a combination of these treatments. Further RCTs would be needed to test any of these interventions. The data acquired in this study will be critical to planning a successful RCT intended to improve the clinical management of combat veterans.

## Conclusions

Retrospective examinations of administrative and medical data indicate that combat-injured service members are at higher risk for the development of adverse long-term health outcomes relative to non-injured combat-exposed service members. However, existing research in this area has been limited. The SPIRIT study represents an important step towards a more comprehensive understanding of the long-term impact of combat injury on physical, mental, and behavioral health. The results will be a foundation for subsequent studies of targeted prevention and interventions in this vulnerable population.

## Supporting information

S1 File SPIRIT protocol. (PDF)
