# Supplementary material for: A study protocol for determining the role of Sympathetic activity in Post-injury outcomes: Impact on sleep and caRdiovascular health InvesTigation (SPIRIT)
Source: PLoS One. 2025 Jul 17;20(7):e0321035. doi: 10.1371/journal.pone.0321035 (PMC12270146; doi:10.1371/journal.pone.0321035)
Supplement: S1 File — (PDF) [file pone.0321035.s001.pdf]

EIRB Protocol Template (Version 1.8)

1.0 General Information

***Please enter the full title of your study:**

Determining the role of sympathetic activity in the impact of combat injury on sleep and cardiovascular outcomes (SPIRIT)

***Please enter the Protocol Number you would like to use to reference the protocol:**

SPIRIT
* This field allows you to enter an abbreviated version of the Protocol Title to quickly identify this protocol.

Is this a multi-site study (i.e. Each site has their own Principal Investigator)?

Yes

Does this protocol involve the use of animals?

○ Yes ◉ No

2.0 Add Site(s)

2.1 List sites associated with this study:

Primary Dept?	Department Name
◆	**R and E** - Uniformed Services University of the Health Sciences (USUHS)

3.0 Assign project personnel access to the project

3.1 *Please add a Principal Investigator for the study:

Stewart, Ian J

Select if applicable

☐ Student ☐ Site Chair
☐ Resident ☐ Fellow

3.2 If applicable, please select the Research Staff personnel:

A) Additional Investigators

Collen, Jacob Fredrick, MD
Sub-Investigator
Haigney, Mark C, MD

Sub-Investigator
Lavender, Jason Matthew, PhD
Sub-Investigator
Solhjoo, Soroosh
Sub-Investigator

B) Research Support Staff

Elisman, Katerina Nicole
Research Coordinator
Fox, Keiko Christine
Research Coordinator
Kalra, Sachi
Research Coordinator
Mains, Autumn Marie
Research Coordinator
Rioux, Stephanie Olivia, BSN
Research Coordinator
Walker, Lauren E
Research Coordinator

3.3 *Please add a Protocol Contact:

Elisman, Katerina Nicole
Fox, Keiko Christine
Haigney, Mark C, MD
Kalra, Sachi
Mains, Autumn Marie
Rioux, Stephanie Olivia, BSN
Stewart, Ian J
Walker, Lauren E

The Protocol Contact(s) will receive all important system notifications along with the Principal Investigator. (i.e. The protocol contact(s) are typically either the Protocol Coordinator or the Principal Investigator themselves).

3.4 If applicable, please select the Designated Site Approval(s):

Add the name of the individual authorized to approve and sign off on this protocol from your Site (e.g. the Site Chair).

4.0 Project Information

4.1 * What department(s) will be associated with this protocol?

	Cardiology
	Sleep

	Nephrology

4.2 * **Is the IRB of record for this study an IRB/HRPP that does NOT use EIRB? If Yes, complete the application according to the IRB/HRPP Determination.**

If your Projects or Protocols are under the oversight of another IRB that does use EIRB, stop this submission and contact the core site and request an invitation as a performing site.

If your Project or Protocol is now being submitted for the first time to an IRB that does use EIRB, continue with this application and answer the questions to be reviewed by the IRB.

Answering yes means the board of record is an IRB that does NOT use EIRB.

○ Yes ◉ No

4.3 * **Is this protocol research, expanded access, or humanitarian use device?**

◉ Yes ○ No

4.4 * **What type of protocol is this?**

- ☑ Behavioral Research
- ☑ Biomedical Research
- ☐ Clinical trial (FDA regulated)
- ☐ Educational Research
- ☐ Expanded Access
- ☐ Humanitarian Use Device (HUD)
- ☐ Psychosocial Research
- ☐ Oral History
- ☐ Other

4.5 Are you conducting this project in pursuit of a personal degree?

○ Yes ◉ No

4.7 * **Is this human subjects research? (As defined by 32 CFR 219) Human subject means a living individual about whom an investigator (whether professional or student) conducting research:**
(i) Obtains information or biospecimens through intervention or interaction with the individual, and uses, studies, or analyzes the information or biospecimens; or
(ii) Obtains, uses, studies, analyzes or generates identifiable private information or identifiable biospecimens.

◉ Yes ○ No

4.8 * **Do you believe this human subjects research is exempt from IRB review?**

○ Yes ◉ No

5.0 Personnel Details

5.1 Does the Principal Investigator have a Permanent Change of Station (PCS) Date or Estimated Institutional Departure Date (EIDD)?

○ Yes ◉ No

5.2 List any Research Team members without EIRB access that are not previously entered in the protocol:

Name: (Last, First, M.I.)	Phone Number:	Email Address:	Associated Institution:
Arnold, Thomas<br>Role on Protocol: Data scientist		thomas.arnold.ctr@usuhs.edu	USUHS

5.3 Are any Contractors or Subcontractors involved in this study? If yes, please list them and describe their role.

○ Yes ◉ No

No records have been added

5.4 Will you have a Research Monitor for this study?

○ Yes
◉ No
○ N/A

6.0 Data/Specimens

6.1 Does the study involve the use of existing data or specimens only (no interaction with human subjects)?

○ Yes ◉ No

7.0 Funding and Disclosures

7.1 Source of Funding:

Funding Source	Funding Type	Amount
: Other<br>DHA	: Other<br>Cooperative agreement	648153

Total amount of funding:

648153

7.2 Do you or any other Investigator(s) have a disclosure of a personal interest or financial nature significant with sponsor(s), product(s), instrument(s) and/or company(ies) involved in this study?

○ Yes ◉ No

All personnel engaged in research must complete and attach a Conflict of Interest (COI) form.

8.0 Study Locations

8.1 Is this a collaborative or multi-site study? (e.g., are there any other institutions involved?)

◉ Yes ○ No

8.2 Study Facilities and Locations:

Institution	Site Name	Site Role	FWA or DoD Assurance Number	Assurance Expiration Date	Is there an agreement?	IRB Reviewing for Site
Navy	Navy Health Research Center (NHRC)	Recruitment	FWA00013516			

Other:

Other Institution Site	Site Role	FWA or DoD Assurance Number	FWA or DoD Expiration Date	Is there an agreement?	IRB Reviewing for Site
No records have been added					

8.3 Are there international sites?

Attach international approval documents, if applicable, when prompted. Note: Ensure local research context has been considered

○ Yes ◉ No

8.4 Is this an OCONUS (Outside Continental United States) study?

○ Yes ◉ No

Select the area of responsibility:

Have you obtained permission from that area of responsibility? (This is a requirement prior to study approval)

○ Yes ○ No

9.0

Study Details

9.1 Key Words:

Provide up to 5 key words that identify the broad topic(s) of your study

Combat Injury, hypertension, cardiovascular disease, sleep disorders, post-traumatic stress disorder.

9.2 Background and Significance:

Include a literature review that describes in detail the rationale for conducting the study. Include descriptions of any preliminary studies and findings that led to the development of the protocol. The background section should clearly support the choice of study variables and explain the basis for the research questions and/or study hypotheses. This section establishes the relevance of the study and explains the applicability of its findings

Background and Significance

Emerging evidence demonstrates that combat casualties are at an increased risk for a wide variety of adverse outcomes, including hypertension (HTN), cardiovascular disease (CVD), sleep disorders, and adverse mental health outcomes such as post-traumatic stress disorder (PTSD).

In 2015, we published the first study to demonstrate an association between the severity of combat injury and subsequent outcomes.1 This study examined the impact of the injury severity score (ISS) on the subsequent diagnoses of HTN and CVD in a cohort of 3,846 service members with combat injury who required care in an intensive care unit (ICU).1 The ISS is a validated, anatomically based scoring system (ranging from 1-75) used to quantify the severity of traumatic injury.2 We found that each five-point increase in the ISS was associated with a 6% increase in the rate of HTN and a 13% increase in the rate of CVD.1 While this work established a relationship between combat injury and subsequent HTN and CVD, there were some limitations. The study lacked a control group of patients that deployed but were not injured and it only included severely injured patients that required ICU-level care.

We sought to address these limitations in our second paper, published in 2021.3 In this study, we randomly selected 10,000 combat-injured subjects from the Department of Defense (DoD) trauma registry. These subjects were then matched to a control group of service members who were deployed, but not injured. After applying inclusion and exclusion criteria, the total N for the study was 17,454. Injury severity was categorized as mild to moderate (ISS 1-24) or severe (ISS≥25). In a multivariable model that adjusted for potential confounders, we found that the rates of HTN were higher for both mild to moderate injury (hazard ratio [HR] 1.14, 95% confidence interval [CI] 1.05-1.24; p=0.002) and severe injury (HR 2.78, 95% CI 2.18-3.55; p<0.001) compared to controls. The rates of CVD were also higher in mild to moderate injury (HR 1.65, 95% CI 1.11-2.37; p=0.013) and severe injury (HR 4.87, 95% CI 2.11-11.25; p<0.001) after adjustment. With a median follow-up time of 8.4 years in this study, these data suggest that combat injury is a risk factor for both HTN and CVD for years after the initial injury. While there is a paucity of evidence in civilian populations, available evidence suggests that traumatic and burn injuries increase the risk for CVD.4 Therefore, research on combat casualties may also have applications for civilian healthcare systems.

Conceptual model

While CVD, HTN, sleep disorders, and adverse mental health outcomes are more common in injured service members, it is important to note that these outcomes do not occur in isolation and that the relationship may be bidirectional. Insomnia has not only been associated with subsequent PTSD, depression, and anxiety,5 but it is also a diagnostic criterion for many mental health conditions.6 In our comprehensive analysis of mental health outcomes in combat-injured service members, we found that insomnia was a risk factor for both PTSD (HR 2.52, 95% CI 2.18-2.93; p<0.001) and depression (HR 2.06, 95% CI 1.85-2.31; p<0.001).5 In contrast, we have also observed that mental health conditions are a risk factor for subsequent insomnia.7 Other considerations are that PTSD has been associated with both activation of the sympathetic nervous system (SNS)8 and inflammation9 in veterans. Furthermore, markers of SNS activation have been associated with both insomnia10 and inflammatory markers.11

Sleep disorders are common among the general population, in the military, and among veterans.12 Insomnia and obstructive sleep apnea (OSA, repetitive collapse of the airway system during

sleep) are the most common sleep disorders. According to the Defense Medical Epidemiological Database, from 2005 to 2019, 393,857 service members were diagnosed with OSA, and 385,537 were diagnosed with insomnia.13 A comprehensive examination of this database revealed that for every 1000 service members in 2014, 75 military medical encounters were for insomnia, and 273 encounters were for mild to severe OSA.14 These figures are derived only from individuals who have sought treatment and likely underrepresent true prevalence rates. Sleep disorders are associated with an increased risk of hypertension, cardiac arrhythmias, and sudden cardiac death. 15–18

Given this body of evidence, the mechanisms that link sleep disorders and mental health conditions to CVD and HTN are likely to be complex and inter-related. As shown in our conceptual model (Figure 1), we hypothesize that combat injury results in: 1) sleep disorders, 2) adverse mental health outcomes and associated behavioral health problems, 3) SNS activation, and 4) inflammation. With regards to the final step in our conceptual framework, subsequent physical health outcomes, poor sleep, mental health symptoms, health behavior problems, SNS activation, and inflammation have all been associated with HTN and CVD. Insomnia was shown to be a risk factor for subsequent HTN in one of our combat casualty cohorts.3 Furthermore, insomnia is a risk factor for HTN19 and treatment of insomnia in patients with depression has been suggested to decrease the subsequent risk of HTN.20 Mental health conditions such as PTSD, have been associated with HTN and CVD in both civilian and military cohorts.3,21–23 Health behavior problems like physical inactivity24 and alcohol use are associated with increased risk of long-term adverse health outcomes.25 SNS activation has also been associated with physical health outcomes including HTN26,27 and CVD risk.28 Lastly, the relationship between inflammation, in particular high sensitivity C-reactive protein (hsCRP), and CVD risk is well established.29,30

Proposed study

The preponderance of studies done in combat-injured subjects are retrospective. Since combat casualties are seen by healthcare providers more frequently than their uninjured counterparts, ascertainment bias may be an issue. Secondly, administrative records do not lend themselves to mechanistic studies. Therefore, the next step is a cross-sectional study to validate the associations observed in the retrospective studies done to date. However, this patient population presents a unique challenge in that they are geographically dispersed, complicating both recruitment and data collection. Given these difficulties, we previously conducted a pilot study to assess the feasibility of such a trial in 119 combat casualties.31 As part of that study, we recruited a subset of combat-injured participants enrolled in Wounded Warrior Recovery Project (WWRP), an ongoing, web-based, longitudinal examination of patient-reported outcomes of

combat-injured service members. Participants were asked to complete a blood draw which is likely perceived as the most inconvenient and participant limiting assessment in any large prospective study. The previous pilot study enrolled 119 subjects from 38 states and the territory of Puerto Rico (Figure 2) and confirmed the feasibility of subsequent trials with this approach. The pilot study demonstrated that we can successfully recruit and obtain data from this dispersed population.

In the context of our successful pilot study and given the clear need to better define the impact of, and the interplay between, HTN, CVD, mental health, SNS activation, and sleep disorders in combat casualties, we propose the present study, “Determining the role of sympathetic activity in the impact of combat injury on sleep and cardiovascular outcomes (SPIRIT).” To further investigate the accumulating evidence suggesting that SNS activation may underlie HTN and be bidirectionally impacted by sleep disorders, we propose to study the association of sleep disorders with electrocardiographic measures and their impact on the risk for increased blood pressure. We propose to use a wearable ambulatory electrocardiogram (ECG) monitor to record one-lead ECGs. In addition to heart rate and heart rate variability (HRV) measures, investigators on this study have used the output from this device to accurately calculate the QT interval and its variability (QTV). Importantly, the ambulatory ECG monitoring is a state-of-the-art method to determine the incidence and nature of arrhythmias, including the percentage of total heartbeats that are due to premature ventricular contractions (PVC), incidence of atrial fibrillation, as well as atrial and ventricular tachycardia. The presence of comorbid obstructive sleep apnea and/or nocturnal hypoxemia, will be evaluated with home sleep apnea testing (HST). Finally, blood pressure will be assessed using a 24-hour ambulatory blood pressure monitor (ABPM), the gold standard for assessing HTN.32 The mean blood pressure and the mean of sleeping blood pressures have been shown to have the most robust association with long-term major cardiovascular events among all blood pressure-related measures.33

The long-term goal of this research effort is to identify the most promising target(s) for an interventional study to prevent excess cardiovascular disease in survivors of combat wounds. These targets could include aggressive treatment of sleep-disordered breathing and hypoxemia by institution of early continuous positive airway pressure (CPAP), more aggressive treatment of hypertension, or more aggressive treatment of PTSD. Further randomized clinical trials (RCT) would be needed to test any of these interventions. The data acquired in this study will be critical to planning a successful RCT intended to improve the clinical management of combat veterans.

9.3 Objectives/Specific Aims/Research Questions:

Describe the purpose and objective(s) of the study, specific aims, and/or research questions /hypotheses

Hypotheses:

1. Severely injured service members (ISS ≥ 15) have higher cardiovascular risk (as defined by the pooled cohort equation and hsCRP) compared to minimally injured service members (ISS ≤ 3).

2. Severe combat injury is associated with increased activation of the SNS and arrhythmias compared to minimal combat injury.

3. Severe combat injury is associated with higher 24-hour mean blood pressures and less nocturnal dipping compared to minimal combat injury.

4. Severe combat injury is associated with greater sleep disorders (insomnia and OSA) compared to minimal combat injury.

5. Mental health conditions (PTSD and depression) are associated with SNS hyperactivity, sleep disorders, and cardiovascular risk.

Objective 1: Collect robust ECG and blood pressure data at a singular time point from a cohort of severely injured (ISS ≥15; n=100) and minimally injured (ISS ≤ 3; n=100) current and former service members enrolled in the WWRP.

Objective 2: Examine the associations between ECG variables, sleep, mental health, and physical health outcomes including elevated blood pressure.

9.4 Study Design:

Describe study design in one to two sentences (e.g., prospective, use of existing records/data /specimens, observational, cross-sectional, interventional, randomized, placebo-controlled, cohort, etc.). Specify the phase – Phase I, II, III, or IV – for FDA-regulated investigational drug research

A prospective, cross sectional study of two groups of participants (100 participants per group) who were injured in combat, enrolled in the WWRP and have previously agreed to be contacted for future research. This study will further investigate the accumulating evidence suggesting that SNS activation may underlie HTN and be bidirectionally impacted by sleep disorders, we propose to study the association of sleep disorders with electrocardiographic measures and their impact on the risk for increased blood pressure.

9.5 Target Population:

Describe the population to whom the study findings will be generalized

Two groups of participants (100 participants per group) who were injured in combat, enrolled in the WWRP, and have previously agreed to be contacted for future research will be referred from the WWRP. The first cohort will be composed of severely injured participants defined as ISS ≥ 15. The second cohort will be a group of participants minimally injured, defined as ISS ≤3.

9.6 Benefit to the DoD:

State how this study will impact or be of benefit to the Department of Defense

The Defense Health Agency 2017 Initial Capabilities Document for Cardiovascular Care identified the following "gap" area as one of the five most important: "Lacking sufficient understanding of the accelerating effects of trauma and wounds on [CVD] development as well as the effects of CVD on [service members] injured or wounded in combat."160 There is no more salient problem in military cardiology than understanding and preventing the excess cardiovascular morbidity experienced by wounded warriors. The data sought by this study will inform Clinical Practice Guidelines for combat veterans with respect to the treatment of sleep disorders and hypertension. These data will inform prospective clinical trials testing therapies to reduce hypertension and improve sleep quality in our Wounded Warriors in order to mitigate the excess in cardiovascular disease seen in survivors of combat injury.

10.0 Study Procedures, Data Management, and Privacy

10.1 Study Procedures:

Describe step-by-step how the study will be conducted from beginning to end

Research Strategy and Feasibility:

Participant Recruitment

We propose a prospective, cross sectional study of two groups of participants (100 participants per group) who were injured in combat, enrolled in the WWRP and have previously agreed to be contacted for future research. The first cohort will be primarily composed of severely injured participants with an ISS ≥ 15. If enrollment targets are not met with recruiting in this range, then the study team will recruit participants with an ISS ≥ 11. The second cohort will be a group of participants who were minimally injured, defined as ISS ≤ 3. NHRC staff will use DoD SAFE (https://safe.apps.mil/) to send selected research staff at MiCOR the encrypted files containing names, contact information (phone numbers), injury characteristics, and the ISS of eligible WWRP participants. Eligible WWRP participants are those who 1) have indicated that they are open to being contacted about future research and 2) have an ISS within eligible ranges for this study.

Research staff at MiCOR will contact study candidates via phone call (see Appendix 1) regarding their willingness to participate in study activities (e.g.,using wearables for seven consecutive days and providing laboratory samples). Research staff will attempt to contact study candidates no more than three times, with one to fourteen days between each attempt. If directed to voicemail when calling a study candidate, research staff will identify who they are, where they are calling from, provide a call-back number, and broadly state that they are contacting the study candidate for a research study (see Appendix 1). If an individual is interested in participating, study staff will discuss, or schedule a time to discuss, with the participant over telephone or HIPAA-compliant video conference call to complete the consent process. If the individual wishes to participate, the consent and HIPAA documents will be signed in the USU REDCap. See Appendix 1 for recruitment phone call and voicemail scripts.

Study Procedures

All study test results will be shared with participants if requested, and any clinically actionable findings will be brought to participants' attention.

Participants will be asked to visit their local selected lab for a single blood and urine draw to collect laboratory measurements. The research coordinators can help participants identify where the closest selected lab is to their home and assist participants in scheduling as needed. Research coordinators will also assist in confirming the order for the participant is present in the selected lab's system prior to the participant's visit. The selected lab will then send the collected sample to the analyzing lab. Once analysis is complete, the study team will be sent the results for entry into the study database. All lab results will be reviewed by a board certified physician for clinical relevance. Any clinically significant and actionable results will be disclosed to the participant directly with recommended follow up. Labs should be completed within four months of enrollment. Research staff will contact participants up to three times to remind them to complete a lab visit.

Research staff will offer instructions on completing all survey measures in REDCap and share the appropriate log in/ link information for participants to complete.

Research staff at MiCOR will mail the ambulatory ECG monitor to participants at their preferred address, along with instructions and materials for mailing the devices back to the study team at USUHS or the respective vendor accordingly. The ambulatory ECG monitor vendor will interpret results of the test and send results to research staff at MiCOR.

HSTs will be mailed to participants, who are not currently undergoing CPAP treatment, by the supplier, along with instructions for return shipment. The vendor will interpret results of the HST

and send results to research staff at MiCOR. For any device being returned, study staff will ensure that the participant's name and mailing address are not included in the return address. The study site's information will be the return address.

24-hour ABPM will be mailed to participants by the supplier, along with instructions for return shipment. The vendor will provide results to research staff at MiCOR.

To compensate participants for their time and inconvenience, they will be given a $50 reimbursement for the lab draw, $50 when the ECG, ABPM and HST are returned followed by $50 when all surveys are completed. This is a total reimbursement of up to $150 per participant.

Study Variables/ Data Collection

Electrocardiogram (ECG): The ECG will be recorded using an ambulatory ECG monitor placed on the sternum. The participants will be instructed on how to administer the monitor and what data needs to be shared with the study team (time of inserting the battery in the device which initiates recording, etc). The ECG monitor is capable of continuously recording the ECG signal for up to 14 days, although compliance falls significantly after seven days. For assessing the frequency of arrhythmias, which are stochastic events, we will record the ECG signal continuously for seven days. At the conclusion of the wear period, the participant will mail the device back to the ECG service provider who will then extract the data and generate a results report for the study team. After the 7 days of wear for the ambulatory ECG, it will take approximately a week from when the CAM arrives at the service provider. Once the results are available in the secure portal, they will then be reviewed by a board certified physician for clinical relevance. Any clinically significant and actionable results will be disclosed to the participant directly with recommended follow up. If the completed recording does not reach the minimum quality needed for proper interpretation, the participant will be asked to repeat the recording.

The frequency of episodes of atrial fibrillation or atrial flutter lasting longer than five minutes, the density of PVCs (percentage of total heartbeats that are PVCs), and the frequency of ventricular tachycardia will be captured from these reports for hypothesis 2.   Heart rate, HRV measures, and QTV measures will be automatically calculated for each 5-min epoch of the ECG recordings. HRV time-domain measures include standard deviation of beat-to-beat intervals (SDNN), root mean square of successive differences (RMSSD), and frequency domain measures including low frequency power (LF, 0.04 – 0.15 Hz), high frequency power (HF, 0.15 – 0.4 Hz), and LF/HF ratio. QTV measures include log-transformed QT variance (logQTv) and beat-to-beat QT variability index (QTVI).

QT interval measures are semi-automatically calculated using a template-matching algorithm. 34,35 At least one QT template is generated for each 24-hour segment of the ECG signal. The template is the average of QT waveforms extracted from a 5-min epoch chosen using a scoring system that incorporates measures including <5% PVCs and low levels of change in RR intervals during each epoch. Ectopic beats, and the beats following them, are excluded. The entire T-wave template is fitted to each beat in the epoch and therefore the method is robust against noise occurring on the isoelectric line or small changes in the T wave shape. We have used this algorithm in analyzing seven independent datasets34,36–40 and have confirmed a very high degree of inter-individual agreement.41 QTVI is defined as the ratio between the normalized variances of the QT interval and heart rate, and is calculated as $QTVI = \log[(QTv/QTm^2)/(HRv/HRm^2)]$, where QTm is mean QT, HRm is mean heart rate, and HRv is the heart rate variance.

Sleep assessment: Sleep will be monitored using a home sleep study test device (HST). Participants currently undergoing CPAP treatment will be excused from the HST portion of the study. The HST will be mailed directly to the participant to administer at home. The HST should be worn during sleep during the second or third study day (the day following ECG placement). The participants will be instructed on how to administer the monitor and what data needs to be shared with the study team (time began recording, etc). At the conclusion of the wear period, the participant will mail the device back to the HST service provider who will then extract the data and generate a results report for the study team. The results will then be reviewed by a board certified physician for clinical relevance. Any clinically significant and actionable results will be disclosed to the participant directly with recommended follow up. If the completed recording does not reach the minimum quality needed for proper interpretation, the participant will be asked to repeat the recording.

Ambulatory Blood pressure: Blood pressure will be measured using 24-hour ABPM. The ABPM will be mailed to the participant by the service provider to be worn during the 24-hour period following the HST, in parallel with the Ambulatory ECG Monitor. The participants will be instructed on how to administer the monitor and what data needs to be shared with the study team (time began recording, etc). The ABPM device will be shipped back to the service provider by the participant where the raw data will be uploaded to a secure portal and shared with the study team. The mean blood pressures for the entire 24 hours and for the time of sleep will be

computed. Nocturnal dipping, the drop in blood pressure during sleep, will also be assessed. The results will then be reviewed by a board-certified physician for clinical relevance. Any clinically significant and actionable results will be disclosed to the participant directly with recommended follow up. If the completed recording does not reach the minimum quality needed for proper interpretation, the participant will be asked to repeat the recording. Of note, the ABPM should not be worn at the same time as the sleep assessment monitor. Participants will also receive this notice.

Inflammatory marker: The participants will undergo measurement of hs-CRP as part of the blood collection and analysis.

Mental health assessments: The severity of PTSD symptoms will be assessed by the PCL-5 20-question survey.42 The severity of depressive symptoms will be measured by the eight-question PHQ-8 survey.43 These will be administered through the USU REDCap system directly to participants. As these are screening measures, not official diagnostic tools and do not assess acute risk, results will not be returned to participants. A list of resources will be given to all participants from the study team.

For PHQ-8, the cut-off score is 10 or higher- below that is considered mild or absent depression symptoms. For the PCL-5 the literature supports a cut-off score of 33. While these scores are cut-offs- these measures are not diagnostic tools but screening measures that provide scores that may be suggestive of the presence of mental health disorders (PTSD, depression, dissociative disorders).

Severity of dissociative experiences will be assessed using the Brief Dissociative Experience Scale (DES-B). Participant-reported sleep characteristics will be assessed using the Pittsburgh Sleep Quality Index (PSQI), the Epworth Sleepiness Scale (ESS), the Insomnia Severity Index (ISI), and the Berlin Sleep Questionnaire. There is no well-established cut-off score for the DES-B since it is meant to be a continuous symptom measure rather than a screener.

A study staff member will review the scores for the PHQ-8 and the PCL-5. If a participant receives a score in the cut-off range, then a study staff member will reach out to the participant to disclose the score and recommend they report these results to their primary care provider or see a mental health provider. A Mental Health referral list will also be provided to them.

Body mass index (BMI): Each participant’s height and weight will be measured and used to calculate BMI at the laboratory where their labs are drawn.

Laboratory measurements: Participants will be asked to visit their local selected lab for a single blood and urine draw to collect laboratory measurements. At the blood draw, seven tubes with 5mL each will be collected. In total, 35mL, or a little more than two tablespoons of blood, will be drawn. LabCorp will also collect height, weight, waist circumference, and blood pressure. Blood will be drawn for the following tests: hsCRP, Lipid panel, Hgb A1C, Comprehensive Metabolic Panel (*Includes: Alanine aminotransferase (ALT/SGPT); albumin:globulin (A:G) ratio; albumin, serum; alkaline phosphatase, serum; aspartate aminotransferase (AST/SGOT); bilirubin, total; BUN; BUN:creatinine ratio; calcium, serum; carbon dioxide, total; chloride, serum; creatinine, serum; eGFR calculation; globulin, total; glucose, serum; potassium, serum; protein, total, serum; sodium, serum), NT-proBNP, and Cystatin C. Urine will be collected to test urine microalbumin creatinine ratio. The research coordinators can help participants identify where the closest selected lab is to their home and assist participants in scheduling as needed. Research coordinators will confirm the lab tests are ordered for each participant in the selected lab’s system prior to the participant’s visit. The selected lab will then send the collected sample to the analyzing lab. Once analysis is complete, the results will be sent to the study team for entry into the RedCap study database. Biological samples will be destroyed by the participating lab after all protocol required testing has been performed. Lab results will be reviewed as soon as they are available and will be evaluated by a board certified physician for clinical relevance. Review of the results depends on the processing time of the selected lab. Participants will be notified after the results are reviewed and any clinically significant and actionable results will be disclosed to the participant directly with recommended follow up. Labs should be completed within four months of enrollment. Research staff will contact participants up to three times to remind them to complete a lab visit.

It will be made clear to the participants that many of the health measures collected will not be reviewed in real-time so any clinical actionable results that are reviewed at a later point, will be disclosed to the participant directly with recommended follow up.

Limitations & Alternative Methods

A limitation to any study regarding a cross-sectional study is that it will be associative. To prove causation, we would need to either increase or decrease SNS activity and assess the impact on blood pressure. Increasing SNS activity with drugs has been attempted in the past but is now

considered unethical given the proven risk of provoking arrhythmias and myocardial ischemia. Reducing SNS activity pharmacologically is less controversial from a human use perspective, but performing such a drug trial or surgical intervention presumes that the SNS is hyperactive, the very hypothesis this study seeks to test.

10.2 Data Collection:

Describe all the data variables, information to be collected, the source of the data, and how the data will be operationally measured.

Demographics (collected through USU REDcap or verbal interview with participant as applicable)

- Age at time of survey completion
- Date of birth
- Sex
- Race and ethnicity
- Rank (current rank if active duty or highest rank for veterans)
- Marital status
- Mailing address
- Socioeconomic status (income level and highest level of education)
- Deployments (number, length, and location)

Injury Characteristics and additional demographics (obtained from WWRP)

- Age
- Year of injury
- ISS
- Mechanism of injury
- Amputations
- TBI history

Surveys (Administered through USU REDCap as applicable)

- PTSD checklist for DSM-5 (PCL-5)
- Brief dissociative experience scale (DES-B)
- Patient Health Questionnaire 8 (PHQ-8)
- Pittsburgh Sleep Quality Index (PSQI)
- Epworth Sleepiness Scale (ESS)
- Insomnia Severity Index (ISI)
- Berlin Sleep Questionnaire

History and Demographics (collected through USU REDcap or verbal interview with participant as applicable)

- Smoking and drug use (smoking is part of Pooled cohort equation)
- Personal and family medical history of premature cardiovascular disease
- Review of medications and past medical history

Laboratory measurements (collected from partner lab- LabCorp)

- Blood Pressure, Height, weight, BMI, and Waist Circumference
- hsCRP
- Lipid panel
- Hgb A1C
- Albumin/creatinine ratio, random urine
- Comprehensive Metabolic Panel (*Includes: Alanine aminotransferase (ALT/SGPT); albumin:globulin (A:G) ratio; albumin, serum; alkaline phosphatase, serum; aspartate aminotransferase (AST/SGOT); bilirubin, total; BUN; BUN:creatinine ratio; calcium, serum; carbon dioxide, total; chloride, serum; creatinine, serum; eGFR calculation; globulin, total; glucose, serum; potassium, serum; protein, total, serum; sodium, serum)
- Cystatin C with eGFR
- NT-proBNP

Wearables

- Ambulatory Electrocardiogram (ECG) Monitor
- 24-hour Ambulatory Blood Pressure Monitor (ABPM)
- Home Sleep Test (HST)

10.3 At any point in the study, will you request, use, or access health information in any form, including verbal, hard copy and electronic?

(•) Yes ( ) No

10.4 Review the definitions below and respond to the following two questions. If you are not sure of the answers, email DHA.PrivacyBoard@mail.mil for assistance. The *Military Health System (MHS)* is defined as all DoD health plans and DoD health care providers that are organized under the management authority of, or in the case of covered individual providers, assigned to or employed by, the Defense Health Agency (DHA), the Army, the Navy, or the Air Force *MHS workforce members* are employees, volunteers, trainees, and other persons whose conduct, in the performance of work for the MHS, is under the direct control of the MHS, whether or not they are paid by the MHS. *MHS business associates* are persons or entities that provide a service to the MHS and require protected health information (PHI) to provide the service.

Are you an MHS workforce member?

( ) Yes, I am an MHS workforce member
(•) No, I am not an MHS workforce member

10.5 Have you consulted with an MHS data expert to determine the data elements required for your study?

Consulting with a data expert often saves time later in the compliance process because the data expert can advise on the data available in the numerous MHS information systems, the quality of that data and the methods for encrypting and collapsing data. To schedule a consult with an MHS data expert, send an email to: (**DHA.PrivacyBoard@mail.mil**)

- ○ Yes, then complete the questions below according to the data consult
- ◉ No, then complete the questions below according to the best of your knowledge

10.6 Indicate how you will request data from the MHS. Select all that apply.

- ☐ Talking with MHS health care providers or MHS health plans about specific research participants
- ☐ Obtaining MHS hard copy records specific to research participants
- ☐ Obtaining data from an MHS information system(s)

10.7 If you are obtaining data from an MHS information system(s), indicate whether you plan to receive a data extract or whether you plan to access an MHS information system directly to create a data set.

A data extract is when the MHS or a contractor provides the data set directly to the researcher. When receiving a data set through data extract, the researcher may indicate whether the data elements should be provided as is, encrypted or collapsed. In contrast to a data extract, access to an information system means that the researcher may directly access an MHS information system and create a data set for the research study

- ☐ Data Extract
- ☐ Access

10.8 Do you intend to request de-identified data from the MHS in your research study?

There are different two methods for de-identifying data pursuant to HIPAA:
1) Safe Harbor Method: Removing all of the identifiers listed in Table 1 below, provided that the researcher does not have actual knowledge that the remaining data can be used alone or in combination with other information to identify the individual who is the subject of the information
2) Statistical Method: An expert, with appropriate knowledge of and experience with generally accepted statistical and scientific principles and methods for rendering information not individually identifiable, determines that the data is not individually identifiable

○ Yes ◉ No

10.9 Indicate the MHS information system(s) from which you will seek to obtain data

If you do not know which system(s) contains the data elements you need, refer to the Guide for DoD Researchers on Using MHS Data or request guidance from an MHS data expert at: **DHA. PrivacyBoard@mail.mil**.

Below is a list of commonly used MHS systems. If the system from which you seek to obtain data is not listed below, list the name of the system in the “Other MHS Systems” category below
PHI Systems:

MHS Information System	Requesting Data
No records have been added	

PII-Only Systems:

MHS Information System	Requesting Data
No records have been added	

De-Identified Data & Other Systems:

Information System	Requesting Data
No records have been added	

10.10 Do you intend to merge or otherwise associate the requested data with data from any sources outside of the MHS, including other DoD systems that are not part of the MHS?

- ○ Yes, will merge data
- ○ No, will not merge data

10.11 Indicate the data elements about research participants or relatives, employers, or household members of the research participants that you will request from MHS hard copies or from MHS information systems.
If you will merge data, also indicate non-MHS data elements about research participants or relatives, employers, or household members of the research participants that you will have access to in any form or medium.

Direct and Indirect Identifiable Data Elements	DHA Hard Copies	DHA Data Elements to be Accessed	DHA Data Elements Verbal	Extracted DHA Digital Data	Downloaded DHA Digital Data	Non-DHA Hard Copies or Digital
1. Names	☐	☐	☐	☐	☐	☑
2. Postal address with only town, city, state, and zip code	☐	☐	☐	☐	☐	☐
3. Postal address with all geographic subdivisions smaller than state, including street address, city, county, precinct, zip code and their equivalent geocodes, except for the initial three digits of a zip code if, according to the current publicly available data from the Bureau of Census: 1) the geographic unit formed by combining all zip codes with the same three initial digits contains	☐	☐	☐	☐	☐	☐

more than 20,000 people; and 2) the initial three digits of a zip code from all such geographic units containing 20,000 or fewer people is changed to 000						
4. Dates including all elements (except year) directly related to an individual, including birthdate, admission date, discharge date, and date of death	☐	☐	☐	☐	☐	☑
5. Ages over 89 and all elements of dates (including year) indicative of such age, unless you will only request a single category of “age 90 or older”	☐	☐	☐	☐	☐	☐
6. Telephone Numbers	☐	☐	☐	☐	☐	☑
7. Fax Numbers	☐	☐	☐	☐	☐	☐
8. Email Addresses	☐	☐	☐	☐	☐	☐
9. Social Security Numbers	☐	☐	☐	☐	☐	☐
10. Medical Record						

Numbers (MRN) (including record ID)	☐	☐	☐	☐	☐	☐	
11. Health Plan Beneficiary Numbers (including DEERS ID, Electronic Data Interchange Personal Identifier (EDIPI) or Number (EDIPN))	☐	☐	☐	☐	☐	☐	
12. Account Numbers	☐	☐	☐	☐	☐	☐	
13. Certificate /License Numbers	☐	☐	☐	☐	☐	☐	
14. Vehicle identifiers and serial numbers, including license plate numbers	☐	☐	☐	☐	☐	☐	
15. Device identifiers and serial numbers	☐	☐	☐	☐	☐	☐	
16. Web Universal Resource Locators (URLs)	☐	☐	☐	☐	☐	☐	
17. Internet Protocol (IP) address numbers	☐	☐	☐	☐	☐	☐	
18. Biometric identifiers, including finger and voice prints	☐	☐	☐	☐	☐	☐	

19. Full-face photographic images and any comparable images	☐	☐	☐	☐	☐	☐
20. Any other unique identifying number, characteristic, or code (including non-military provider IDs)	☐	☐	☐	☐	☐	☑
21. Free Text Fields	☐	☐	☐	☐	☐	☐

If you are obtaining SSNs, provide a justification as to why and explain why a substitute cannot be used.

Due to guidelines stated within DoDI 1000.30, Reduction of SSN Use within DoD, the reduction or elimination of SSN usage must occur wherever possible. If SSNs are required to complete the project, the PI must provide a justification and explanation as to why a substitution cannot be used.
For example:

- If alternatives to SSN (e.g., EDIPNs or pseudo person IDs) are sufficient in other instances, will those alternatives to SSN usage be sufficient to respond to Congressional inquiries and /or Senior DoD stakeholders inquiries?
- Are alternatives to SSN used first?
- Are those alternatives to SSN insufficient to combine data from multiple data sources? Is the issue that some individuals do not possess alternatives ID numbers and SSN is the only way to identify them?

a. Will you receive or obtain health information?

> ***Note:*** *If you indicate you are not receiving health information, the answer must be consistent with the DHA data source. For a non-health information data request, if you are a non-MHS employee or non-MHS business associate, you may not access an information system that has PHI or LDS. For both MHS and Non-MHS employees and MHS business associates, you may* ***NOT*** *include data elements in the above table on: 1) lines 10 or 11, 2) line 21 if the free text field comes from a PHI or LDS system, and 3) lines 12, 13, or 18 if the account numbers, certificate and license numbers, biometric data, or any other data elements are health information created or received by an MHS health care provider, health plan, or business associate in relation to the physical or mental health or condition of an individual or payment for health care.*

○ Yes, I will receive or obtain health information

◉ No, I will not receive or obtain health information

b. If no data elements were checked in the above table, is it possible that the requested DHA data is or will be identifiable because of any unique data elements, triangulation, or small cell size?

☐ Data elements were checked in the above table, STOP HERE.

NOTE: *A unique data element includes any unique features that alone are not identifiable but that could be used to identify an individual within the context of other information, such as any type of code (such as diagnosis or procedural), rank of general or admiral, gender, or race. Triangulation means using different data elements that when combined can be used to identify an individual, such as including the above lists of unique data elements in a data set. Determining whether an individual is identifiable through triangulation requires consideration of all data elements in combination. Within the military, the use of rank and/or diagnosis code, procedural codes, or any other code that changes on a predictable basis, increases the possibility of identification. Small cell size means that there is only a small number of eligible individuals that satisfy the category description. Department of Defense Manual 6025.13, Medical Quality Assurance and Clinical Quality Management in the Military Health System MHS, provides that the threshold for de-identifying data within the MHS requires a cell size of three, but also states that the de-identification standards must meet the DoD implementation of the HIPAA Privacy Rule. Centers for Medicare and Medicaid also gives guidance on small cell size stating that no data cell less than 11 may be published or displayed. However, the Office for Civil Rights' OCR, which is the official regulatory office for the HIPAA Privacy Rule, provides that OCR does not designate a universal value for small cell size in accordance with the de-identification standard; instead, the cell size should be set at a level that is appropriate to mitigate risk of identification by the anticipated recipient of the data set. This means that a cell size of 3 or 11 may not meet the HIPAA Privacy Rule requirements if the cell size level does not appropriately mitigate risk of identification by the anticipated recipient of the data set.*

Note: If dates are altered as a means of de-identifying the data, diagnosis and procedural codes need to be rolled-up or collapsed. If dates are provided "as time between events," the roll-up is not necessary.

- [ ] Yes, the DHA data will become identifiable
- [ ] No, the DHA data will not become identifiable

10.12 Do you believe it is possible for the MHS data to become identifiable because of triangulation, a small cell size, or any unique data element(s)?

Triangulation means using different data elements that are not themselves identifiable but that when combined can be used to identify an individual. For example, triangulation would use rank and race together to determine the identity of an individual with a particular health condition.

Small cell size means that there is only a small number of eligible individuals that satisfy the category description. Guidance for acceptable cell size is available from the Centers for Medicare and Medicaid Services. For example, the rank category of four star generals with a particular diagnosis may be less than 30, so the rank category may need to be expanded to include lower ranks.

A unique data element includes any unique features that are not explicitly enumerated in the categories of data in rows 1 – 20 of the table above (in Section 10.10), but that could be used to identify an individual. Unique data elements include characteristics that are not themselves identifying, such as the rank of general or admiral, or a race or gender, but within the context of other information could be identifiable.

- ○ Yes, I believe there is a reasonable possibility the MHS data will become identifiable
- ○ No, I believe there is no reasonable possibility the MHS data will become identifiable

10.13 Have you completed and uploaded an appropriate HIPAA document (i.e. HIPAA Authorization will be obtained or Waiver/alteration of HIPAA Authorization is being requested)?

- ◉ Yes
- ○ No
- ○ N/A

If yes, please check which one.

- [x] HIPAA Authorization
- [ ] HIPAA Waiver (Full or Partial)
- [ ] Other (please provide copies when uploading Other Study Documents)

10.14 Managing Data (Data Management and/or Sharing Plan) and/or Human Biological Specimens for this Study:

Include in this section the plan for acquiring data (both electronic and hard copy), access during the study, data/specimen storage and length of time stored, shipment/transmission, and the plan for storage and final disposition at the conclusion of the study. Describe any data agreements in place for accessing data within and/or outside of your institution (e.g., Data Sharing Agreement, Data Use Agreement, Business Agreements, etc.)

Data management:

The study participants will be assigned a unique numerical identifier and all applicable data at USU will be coded by using only this study number. Necessary HIPAA identifiers (name, date of birth, and mailing address as applicable) will be shared with the applicable vendors for labs and wearable devices. If applicable, the master subject ID log (hard copy version), hard copy CRFs, ICF/HIPAA and source documents will be stored in a locked cabinet within a secured office for the duration of the study. All electronic ICF/ HIPAA/ CRFs will be stored within the selected USU REDCap database system according to data safety and security standards set forth by the University. Upon conclusion of the study and completion of data analysis the master subject ID log will be destroyed. Source files and Consent Forms/HIPAA Authorizations will be kept for the required number of years following study closure. Only study team members who have been trained to protect patient privacy will have access to the data files. Only the study nurse, study coordinator, and PI will have access to the participant ID log identifying participant PPI and study code number.

Electronic files will be maintained in the USU REDCap electronic data capture system for the duration of the study for use in future research studies by authorized researchers. The data protection for privacy and confidentiality described in the ICF would apply to any future use of stored data. Any future research will be IRB approved or have appropriate institutional review.

All data will be destroyed after scientific utility is no longer relevant. Data will become deidentified upon study completion. Following the required time for documents to be stored, all hard copy documents (if any) will be shredded and sent for incineration at a secure facility. The electronic data will be deleted according to applicable policy and 21 CFR guidelines following final publication or until scientific utility is no longer relevant, whichever is longer. All applicable data will be coded and maintained in a HIPAA compliant manner.

Is this a data repository?

○ Yes ◉ No

10.15 Managing Data (Data Management and/or Sharing Plan) and/or Human Biological Specimens for Future Research:

If the study involves collecting, storing, or banking human specimens, data, or documents (either by the Investigator or through an established repository) for FUTURE research, address. How the specimens/data will be used, where and how data/specimens will be stored (including shipping procedures, storage plan, etc.), whether and how consent will be obtained, procedures that will fulfill subjects' request as stated in the consent, whether subjects may withdraw their data/specimens from storage, whether and how subjects may be recontacted for future research and given the option to decline, whether there will be genetic testing on the specimens, who will have access to the data/specimens, and the linkage, the length of time that data/specimens will be stored and conditions under which data/specimens will be destroyed.

The study participants will be assigned a unique numerical identifier and all applicable data at USU will be coded by using only this study number. Necessary HIPAA identifiers (name, date of birth, and mailing address as applicable) will be shared with the applicable vendors for labs and wearable devices. If applicable, the master subject ID log (hard copy version), hard copy CRFs, ICF/HIPAA and source documents will be stored in a locked cabinet within a secured office for the duration of the study. All electronic ICF/ HIPAA/ CRFs will be stored within the selected USU REDCap database system according to data safety and security standards set forth by the University. Upon conclusion of the study and completion of data analysis the master subject ID log will be destroyed. Source files and Consent Forms/HIPAA Authorizations will be kept for the required number of years following study closure. Only study team members who have been

trained to protect patient privacy will have access to the data files. Only the study nurse, study coordinator, and PI will have access to the participant ID log identifying participant PII and study code number.

Electronic files will be maintained in the USU REDCap electronic data capture system for the duration of the study for use in future research studies by authorized researchers. The data protection for privacy and confidentiality described in the ICF would apply to any future use of stored data. Any future research will be IRB approved or have appropriate institutional review.

All data will be destroyed after scientific utility is no longer relevant. Data will become deidentified upon study completion. Following the required time for documents to be stored, all hard copy documents (if any) will be shredded and sent for incineration at a secure facility. The electronic data will be deleted according to applicable policy and 21 CFR guidelines following final publication or until scientific utility is no longer relevant, whichever is longer. All applicable data will be coded and maintained in a HIPAA compliant manner.

No biological specimens or results will be stored for future use.

Is this a data repository?

○ Yes ◉ No

11.0 Statistical/Data Analysis Plan

11.1 Statistical Considerations:

List the statistical methods to be used to address the primary and secondary objectives, specific aims, and/or research hypotheses. Explain how missing data and outliers will be handled in the analysis. The analysis plan should be consistent with the study objectives. Include any sub-group analyses (e.g., gender or age group). Specify statistical methods and variables for each analysis. Describe how confounding variables will be controlled in the data analysis

Descriptive Statistics

Data management and analyses will be conducted using Stata (version 15.0, StataCorp). Summary statistics and visualizations will be produced to describe the variables of interest. Study participants will be characterized in terms of demographics and clinical variables using percentages for categorical variables and either means and standard deviations or median and interquartile range for continuous variables. We will assess differences in characteristic distribution by group using t-tests or nonparametric tests, and Chi-square tests (or Fisher's Exact test for small sample sizes) as appropriate. For hypothesis 1 (comparing the cardiovascular risk of participants stratified by combat injury), we will use univariable and multivariable linear mixed-effects models (LMM). For hypothesis 2, univariate and multivariable logistic regression will be used to examine arrhythmia outcomes, while LMM will be used to examine SNS outcomes. These same methods will also be used to examine hypothesis 3 (rates of hypertension and nocturnal dipping), 4 (rates of sleep disorders), and 5 (mental health as a risk factor for subsequent outcomes). In the case of binary outcomes (for example presence or absence of insomnia), logistic regression will be the statistical test of choice. In the case of continuous or ordinal outcomes (e.g. SNS activation and severity of PTSD symptoms), LMM will be the statistical test of choice.

11.2 Sample Size:

n=200 (2 groups of 100)

11.3 Total number of subjects requested (including records and specimens):

200

11.4 If you are recruiting by study arm, please identify the arms of the study and how many subjects will be enrolled in each arm

The first cohort will be composed of severely injured participants with an ISS ≥ 15 (n=100)

The second cohort will be a group of participants who were minimally injured, defined as ISS ≤ 3 (n=100)

11.5 Please provide a justification for your sample size

Power and Sample Size

This study has been powered to detect an unadjusted period prevalence difference of 0.15 between the severely injured (ISS ≥ 15; assumed 25% prevalence) and minimally injured group (ISS ≤3; assumed 10% prevalence), with n=100 per group, two-sided α=0.05, and 80% power.

The sample size of n=100 per group will also be sufficiently powered to detect mean differences of 2.5 in HRV measures, specifically SDNN, with a pooled standard deviation of eight, a moderate effect size of 0.40, with two-sided α=0.05, at 80% power.

11.6 Data Analysis Plan: Complete description: Background, Objectives, Design, Step by Step how the project is going to be done, Data analysis plan:

See section 11.1- Descriptive Statistics plan

12.0 Participant Information

12.1 Subject Population:

Two groups of participants (100 participants per group) who were injured in combat, enrolled in the WWRP, and have previously agreed to be contacted for future research will be referred from the WWRP. The first cohort will be primarily composed of severely injured participants with an ISS ≥ 15. If enrollment targets are not met with recruiting in this range, then the study team will recruit participants with an ISS ≥ 11. The second cohort will be a group of participants minimally injured, defined as ISS ≤3.

12.2 Age Range:

Check all the boxes that apply. if the age range of potential subjects (specimens, records) does not match the range(s) selected, please specify in the text box.

- ☐ 0-17
- ☑ 18-24
- ☑ 25-34
- ☑ 35-44
- ☑ 45-54
- ☑ 55-64
- ☑ 65-74
- ☑ 75+

12.3 Gender:

- [x] Male
- [x] Female
- [x] Other

12.4 Special categories, check all that apply

- [ ] Minors /Children
- [ ] Students
- [ ] Employees - Civilian
- [ ] Employees - Contractor
- [ ] Resident/trainee
- [ ] Cadets /Midshipmen
- [x] Active Duty Military Personnel
- [x] Wounded Warriors
- [ ] Economically Disadvantaged Persons
- [ ] Educationally Disadvantaged Persons
- [ ] Physically Challenged (Physical challenges include visual and/or auditory impairment)
- [ ] Persons with Impaired Decisional Capacity
- [ ] Prisoners
- [ ] Pregnant Women, Fetuses, and Neonates
- [ ] Non-English Speakers
- [ ] International Research involving Foreign Nationals - Headquarters Review is necessary

You must also consider the requirements of DoDI 3216.02, Enclosure 3, paragraph 7.e.

Depending on your intended subjects' status, you may also need to consider the requirements of DoDI 3216.02, Enclosure 3, paragraph 7.e.

12.5 Inclusion Criteria:

Order Number	Criteria
1	Enrolled in WWRP and previously agreed to be contacted regarding future research
2	Able to complete the informed consent process, be mailed study devices and complete laboratory draws within the US
3	Age $\geq$ 18 years

12.6 Exclusion Criteria:

Order Number	Criteria
1	No ISS or $3 < ISS < 15$
2	Unable to complete the informed consent process, be mailed study devices or complete laboratory draws within the US
3	Not enrolled in WWRP or enrolled in WWRP but did not agree to be contacted about future research

13.0 Recruitment and Consent

13.1 Please describe the recruitment process, including how subjects will be identified and selected for the study.

Recruitment Process:

NHRC staff will identify eligible WWRP participants from their existing database. Once identified, NHRC staff will send applicable data to the MiCOR team through secure methods identified above. MiCOR staff will then contact the participants directly as described above.

13.2 Compensation for Participation:

To compensate participants for their time and inconvenience, they will be given a $50 reimbursement for the lab draw, $50 when the ECG, ABPM and HST are returned followed by $50 when all surveys are completed. This is a total reimbursement of up to $150 per participant.

13.3 Please describe the pre-screening process. If no pre-screening, enter Not Applicable in the text editor

N/A

13.4 Consent Process: Revised Common Rule, Section 219.116: General requirements for informed consent, whether written or oral, are set forth in this paragraph and apply to consent obtained in accordance with the requirements set forth in paragraphs (b) through (d) of this section. Broad consent may be obtained in lieu of informed consent obtained in accordance with paragraphs (b) and (c) of this section only with respect to the storage, maintenance, and secondary research uses of identifiable private information and identifiable biospecimens.

Are you requesting a waiver or alteration of informed consent?

○ Yes ◉ No

Please explain the consent process:

Consent Process:
Before any data is collected from participants, a study team member will complete the consent process electronically in USU's REDCap.
1) Informed Consent Form (ICF) process is completed privately over the phone or via a HIPAA compliant video conferencing platform; 2) ICF is reviewed with participant in detail, page by page review; 3) Participant is given ample time to review ICF and ask questions; 4) PI/ Sub-I available to answer questions for participant; 5) Participant receives copy of ICF for their records.

13.5 DoDI 3216.02 requires an ombudsman to be present during recruitment briefings when research involves greater than minimal risk and recruitment of Service members occurs in a group setting. If applicable, you may nominate an individual to serve as the ombudsman.

◉ N/A
○ Propose ombudsman

13.6 Withdrawal from Study Participation:

Explain the process for withdrawal and specify whether or not the subjects will be given the opportunity to withdraw their data their data/specimens in the event they wish to withdraw from the study

Withdrawal from Study:
Study participants are informed in the consent process of their right to withdraw from this study at any time. Participants are instructed to contact either the Principal Investigator or the Research Coordinator in writing or to complete a study withdrawal request form. A verbal request may also be made which will be documented by the study team. Participants will be asked to complete safety follow-up if practicable. Upon receipt of a study withdrawal request form or verbal request to withdraw, study data will be deleted for that participant. If laboratory and diagnostic testing have not been completed at the time of withdrawal, untested specimens will be destroyed. However, the participant will be given the option of allowing the study team to utilize the data that has been collected up until the point of withdrawal.

14.0 Risks and Benefits

14.1 Risks of Harm:

Identify all research-related risks of harm to which the subject will be exposed for each research procedure or intervention as a result of participation in this study. Consider the risks of breach of confidentiality, psychological, legal, social, and economic risks as well as physical risks. Do not describe risks from standard care procedures; only describe risks from procedures done for research purposes

There are less than minimal risks of any harm from participation in this study.

Specimen Collection

The risks of venipuncture include pain, a bruise at the point where the blood was taken, redness, swelling of the vein, infection, and a rare risk of fainting. All efforts will be made by the selected labs to reduce these risks.

Ambulatory ECG Monitor and Ambulatory Blood Pressure Monitor

In addition, participants will be required to wear vital signs and cardiac monitoring devices, entailing minor inconvenience. Wearing monitors may rarely reveal previously undiagnosed cardiac or other medical conditions. A risk for the patient is that they could be distressed by finding out that they have a medical diagnosis. However, discovery of health conditions will give the patient knowledge of their health status and allow them to seek treatments, which could decrease morbidity and mortality.

Home Sleep Study Test

Participants will be asked to perform a home sleep study test as part of this protocol. There may be a minor inconvenience for the wearer including mild discomfort. Wearing monitors may rarely reveal previously undiagnosed medical conditions. A risk for the patient is that they could be distressed by finding out that they have a medical diagnosis. However, discovery of health conditions will give the patient knowledge of their health status and allow them to seek treatments, which could decrease morbidity and mortality.

Data Safety and Security

Another possible risk involves potential breaches of privacy and participant confidentiality, should the data set be acquired by a person or agency outside of this research team. The likelihood of that occurrence will be mitigated by password protection of electronic files and removal of all participants' PHI (except age) prior to any presentations or publication of the data, as detailed in the above description regarding data management and privacy.

Since the home sleep test and ambulatory blood pressure monitor will be sent directly to the participant from the vendor, they will require access to participant name, date of birth, and mailing address. This is strictly for shipping purposes only. The lab where blood is drawn and processed will also need your personal identifiers such as name, date of birth, and home address in order to schedule lab visits and process samples.

Researchers will make every effort to protect your privacy and confidentiality; however, there are risks of breach of information security and information loss.

In addition to the research team members, the following people or organization(s) will be allowed to disclose, use, and receive participant information, but they may only use and disclose the information to the other parties on this list, to you as permitted by law: Any organization participating in an approved research-related data or information exchange in connection with this study.

Necessary HIPAA identifiers will be shared with the applicable vendors for labs and wearable devices in order to carry out necessary study procedures.

• Any organization, laboratory, or vendor participating in an approved research-related data or information exchange in connection with this study.

• Any laboratories, individuals, organizations, and vendors that use your coded samples and health information in connection with this study including USUHS.

• Any federal, state, or local governmental agency that regulates the study.

• U.S. Department of Health & Human Services (DHHS), and Office for Human Research Protections (OHRP) or other government agencies in this or other countries.

• The designated Protocol Review and Monitoring Committees, Institutional Review Boards (IRB), Privacy Boards, Study Monitor, and their related staff that have oversight responsibilities for this study.

Completion of survey measures

Some participants may experience slight discomfort over the topics assessed in the structured clinical interview and certain questionnaires. Others may find cognitive tasks to be challenging or frustrating. However, these procedures rarely pose psychological risks for study participants. A list of resources will be given to all participants from the study team.

Unexpected Findings

It is possible that, based on data collection, participants will meet criteria for diseases like hypertension, diabetes, atrial fibrillation, obstructive sleep apnea, and chronic kidney disease. All labs and wearable data will be reviewed by a board-certified physician. If the study healthcare provider determines that treatment and/or additional diagnostic tests are warranted, the participant will be contacted and informed that they should see their PCM. or visit an emergency room, depending on the acuity of findings as determined by the physician. If the participant is not enrolled in healthcare, they will be encouraged to call VA Health Care at the Health Eligibility Center (HEC) toll free at (855) 488-8440 to determine their eligibility and start the enrollment process. Participants will be provided with a copy of their lab tests upon request.

It will be made clear to the participants that many of the health measures collected will not be reviewed in real-time so any clinical actionable results that are reviewed at a later point, will be disclosed to the participant directly with recommended follow up.

14.2 Measures to Minimize Risks of Harm (Precautions, safeguards):

For each research procedure or intervention, describe all measures to minimize and/or eliminate risk of harms to subjects and study personnel

Described above in Section 14.1

14.3 Confidentiality Protections (for research records, data and/or specimens):

Describe in detail the plan to maintain confidentiality of the research data, specimens, and records throughout the study and at its conclusion (e.g., destruction, long term storage, or banking). Explain the plan for securing the data (e.g., use of passwords, encryption, secure servers, firewalls, and other appropriate methods). If data will be shared electronically with other team members/collaborators outside the institution, describe the method of transmission and safeguards to maintain confidentiality. Explain whether this study may collect information that State or Federal law requires to be reported to other officials or ethically requires action, e.g., child or spouse abuse

As mentioned in the data management section:

The study participants will be assigned a unique numerical identifier and all applicable data at USU will be coded by using only this study number. Necessary HIPAA identifiers (name, date of birth, and mailing address as applicable) will be shared with the applicable vendors for labs and wearable devices. If applicable, the master subject ID log (hard copy version), hard copy CRFs, ICF/HIPAA and source documents will be stored in a locked cabinet within a secured office for the duration of the study. All electronic ICF/ HIPAA/ CRFs will be stored within the selected USU REDCap database system according to data safety and security standards set forth by the University. Upon conclusion of the study and completion of data analysis the master subject ID log will be destroyed. Source files and Consent Forms/HIPAA Authorizations will be kept for the required number of years following study closure. Only study team members who have been trained to protect patient privacy will have access to the data files. Only the study nurse, study coordinator, and PI will have access to the participant ID log identifying participant PII and study code number.

Electronic files will be maintained in the USU REDCap electronic data capture system for the duration of the study for use in future research studies by authorized researchers.

All data will be destroyed after scientific utility is no longer relevant. Data will become deidentified upon study completion. Following the required time for documents to be stored, all hard copy documents (if any) will be shredded and sent for incineration at a secure facility. The electronic data will be deleted according to applicable policy and 21 CFR guidelines following final publication or until scientific utility is no longer relevant, whichever is longer. All applicable data will be coded and maintained in a HIPAA compliant manner.

14.4 Potential Benefits:

Describe any real and potential benefits of the research to the subject and any potential benefits to a specific community or society

If the individuals in the research are considered experimental subjects (per 10 USC 980), and they cannot provide their own consent, the protocol must describe the intent to directly benefit all subjects

There is a theoretical benefit to the participants themselves, as a better understanding of the risk factors for poor long-term outcomes after combat injury could improve the way that we care for these patients in the DoD and VA health systems. The results may also benefit service members injured in current and future conflicts as well as civilians with non-combat trauma. Given the paucity of data on long-term outcomes after combat injury specifically, and trauma generally, the benefits of the proposed work outweighs the minimal potential risks.

14.5 Privacy for Subjects:

Describe the measures to protect subject’s privacy during recruitment, the consent process, and all research activities, etc.

The research team will protect the privacy and security of study participants according to USU research policies and applicable laws and regulations including HIPAA. Data for this project will be obtained only for participants who have signed the informed consent. The protocol will limit access to participant data in compliance with minimum necessary standards. By limiting access to the codes that link participant data to the sources of the specimens/data through physical or cyber procedures, the risk for improper release of data is reduced. Appropriate measures will be taken to implement administrative, technical and physical safeguards to protect participant data. Members of the research team are trained in the protection of participant privacy. Research source documents, data files and the master code linking the participant identity to study ID codes will be maintained for the duration of the study. Consent forms and HIPAA Authorization documents will be kept for the required number of years after the study is closed and then will be destroyed.

Recruitment and consent process will occur over telephone or appropriate virtual environments. Applicable return of results will also occur privately over telephone or other secure virtual platform. In addition, all study participants will be given a study code number by which they will be referred. This will prevent the use of names/ SSN, etc on study documents.

14.6 Incidental or Unexpected Findings:

Describe the plan to address incidental findings and unexpected findings about individuals from screening to the end of the subject's participation in the research. In cases where the subject could possibly benefit medically or otherwise from the information, state whether or not the results of screening, research participation, research tests, etc., will be shared with subjects or their primary care provider. State whether the researcher is obligated or mandated to report results to appropriate military or civilian authorities and explain the potential impact on the subject

Unexpected Findings:

It is possible that, based on data collection, participants will meet criteria for diseases like hypertension, diabetes, atrial fibrillation, obstructive sleep apnea, and chronic kidney disease. All labs and wearable data will be reviewed by a board-certified physician. If the study healthcare provider determines that treatment and/or additional diagnostic tests are warranted, the participant will be contacted and informed that they should see their PCM or visit an emergency room, depending on the acuity of findings as determined by the physician. It will be made clear to the participants that many of the health measures collected will not be reviewed in real-time so any clinical actionable results that are reviewed at a later point, will be disclosed to the participant directly with recommended follow up (please see below for diagnostic criteria that may be identified by the wearable devices or lab evaluations). If the participant is not enrolled in healthcare, they will be encouraged to call VA Health Care at the Health Eligibility Center (HEC) toll free at (855) 488-8440 to determine their eligibility and start the enrollment process. Participants will be provided with a copy of their lab tests upon request.

Diagnostic criteria for potential diseases that may be identified for each device/lab evaluation:

CAM: Arrythmia (atrial fibrilation, atrial flutter, ventricular tachycardia), mean heart rate >100, premature ventricular contraction burden >20%, Atrio-ventricular block > Mobitz type I.

HST: Apnea hypopnea index (AHI) >/= to 5 is consistent with the diagnosis of OSA if the subject has symptoms (defined as habitual snoring, fragmented sleep, excessive daytime sleepiness, or hypertension). AHI >/= 15 events per hour is consistent with the diagnosis of OSA, regardless of symptoms or comorbidities

ABPM: Hypertension is diagnosed when the mean daytime blood pressure is ≥130 mmHg systolic or ≥80 mmHg diastolic.

Blood tests:
- hsCRP: slightly high is 1.0-3.0 mg/L and high is >3.0mg/L. Used to help determine your risk of developing chronic heart disease

- Lipid panel: LDL>190 or triglycerides>1000.
- Hgb A1C: 5.7% to 6.4% indicates prediabetes and 6.5% or more indicates diabetes
-Cystatin C: used in a formula to calculate kidney function. The results of this formula (available at https://www.kidney.org/professionals/kdoqi/gfr_calculator) indicate the level of kidney function. >60ml/min/1.72m2 is considered normal.

The following are normal values in a chemistry panel:
- Albumin: 3.4 to 5.4 g/dL (34 to 54 g/L)
- Alkaline phosphatase: 20 to 130 U/L
- ALT (alanine aminotransferase): 4 to 36 U/L
- AST (aspartate aminotransferase): 8 to 33 U/L
- BUN (blood urea nitrogen): 6 to 20 mg/dL (2.14 to 7.14 mmol/L)
- Calcium: 8.5 to 10.2 mg/dL (2.13 to 2.55 mmol/L)
- Chloride: 96 to 106 mEq/L (96 to 106 mmol/L)
- CO2 (carbon dioxide): 23 to 29 mEq/L (23 to 29 mmol/L)
- Creatinine: used in a formula to calculate kidney function. The results of this formula (available at https://www.kidney.org/professionals/kdoqi/gfr_calculator) indicate the level of kidney function. >60ml/min/1.72m2 is considered normal.
- Glucose: 70 to 100 mg/dL (3.9 to 5.6 mmol/L)
- Potassium: 3.7 to 5.2 mEq/L (3.70 to 5.20 mmol/L)
- Sodium: 135 to 145 mEq/L (135 to 145 mmol/L)
- Total bilirubin: 0.1 to 1.2 mg/dL (2 to 21 μmol/L)
- Total protein: 6.0 to 8.3 g/dL (60 to 83 g/L)
- NT-proBNP:

Age	Male (pg/mL)	Female (pg/mL)
18 y	0 115	0 115
19 to 44 y	0 86	0 130
45 to 54 y	0 121	0 249
55 to 64 y	0 210	0 287
65 to 74 y	0 376	0 301
>74 y	0 486	0 738

Urine test: A ratio of 30-300 signifies microalbuminuria and values above 300 are considered as macroalbuminuria.

15.0 Study Monitoring

15.1 Your study requires either Data and Safety Monitoring Plan (DSMP) or a Data and Safety Monitoring Board (DSMB).

- ○ DSMP
- ○ DSMB
- ○ Both
- ◉ Not Applicable

16.0 Reportable Events

16.1 Reportable Events: Consult with the research office at your institution to ensure requirements are met. Describe plans for reporting unexpected adverse events and unanticipated problems. Address how unexpected adverse events will be identified, who will report, how often adverse events and

unanticipated problems will be reviewed to determine if any changes to the protocol or consent form are needed and the scale that will be used to grade the severity of the adverse event.

Consult with the research office at your institution to ensure requirements are met

• Describe plans for reporting expected adverse events. Identify what the expected adverse events will be for this study, describe the likelihood (frequency, severity, reversibility, short-term management and any long-term implications of each expected event)

• Describe plans for reporting unexpected adverse events and unanticipated problems. Address how unexpected adverse events will be identified, who will report, how often adverse events and unanticipated problems will be reviewed to determine if any changes to the research protocol or consent form are needed and the scale that will be used to grade the severity of the adverse event

1. Adverse events for this protocol are expected to be minimal and limited to events related to device use and blood draw. Adverse events will be assessed by the study staff throughout the screening and enrollment visit. Participants will be instructed to notify the study staff of any other events that occur throughout the study duration. All adverse events, regardless of severity and/or relationship to the protocol, will be documented and reported to the USU IRB from the time of informed consent until the completion of all study procedures. The IRB meets regularly to review research plans and adverse events. The IRB has the authority to request modifications to the protocol or halt the study if the safety of the participants is not assured. Serious adverse events are defined by the FDA as any untoward medical occurrences that: (1) result in death, (2) are life threatening, (3) require (or prolong) hospitalization, (4) cause persistent or significant disability /incapacity, (5) result in congenital anomalies or birth defects, or (6) are other conditions which, in the judgment of the investigators, represent significant hazards. Serious adverse events will be reported by the PI verbally and in writing as soon as possible to the IRB, with a maximum of 7 calendar days for death or life-threatening adverse events, and within 15 days for all other serious adverse events. Adverse events are defined by the FDA as any unfavorable and unintended diagnosis, symptom, sign (including an abnormal laboratory finding), syndrome or disease which either occurs during the study, having been absent at baseline, or, if present at baseline, appears to worsen.
2. UPIRTSOs, are defined as unexpected AEs and SAEs that are possibly related to research study participation in the opinion of the PI AND place subjects or others at a greater risk of harm that was previously known or recognized in the protocol. UPIRTSOs must be reported to the IRB via email or telephone within 24 hours of discovery and a written follow-up report must be provided within 5 business days. Expected reportable events and events that are not related to study participation are reported on the Adverse Event Log and will be reviewed at either the annual Continuing Review (CR) Progress Report or at the close of the study. CR is generally performed on a 12-month cycle. More frequent Progress Reports may be required at the discretion of the IRB. The investigator is required to make the determination as to whether the deviation meets the criteria for an unanticipated problem involving risks to subjects or others. The IRB Chair or IRB staff member shall also make the determination if the protocol deviation meets the definition of an unanticipated problem involving risks to participants or others. If the IRB Chair or IRB Staff member determines and documents that the deviation is an unanticipated problem involving risks to subjects or others, or the deviation resulted from serious or continuing noncompliance, the IRB staff member shall place the deviation on the agenda of the next available IRB meeting for review. If the IRB Chair or IRB Staff member determines and documents that the deviation is not an unanticipated problem involving risks to subjects or others, the IRB Chair or staff member shall acknowledge the submission and complete the review through an administrative review procedure.

17.0 Equipment/non-FDA Regulated Devices

17.1 Does the study involve the use of any unique non-medical devices/equipment?

○ Yes ◉ No

18.0 FDA-Regulated Products

18.1 Will any drugs, dietary supplements, biologics, or devices be utilized in this study?

- [ ] Drugs
- [ ] Dietary Supplements
- [ ] Biologics
- [x] Devices
- [ ] N/A

18.3 Device Details:

- [x] Are device(s) in this research being used in accordance to the approved labeling?
- [ ] Are device(s) in this research being used in a manner other than its approved labeling?

When adding a device indicate in the details section of the device if the use is either used in accordance to the approved labeling or in a manner other than it's approved labeling

View Details	Device Name
⊟	The Bardy Dx Carnation Ambulatory Monitor (CAM)

Manufacturer/Supplier of Device	Bardy
Where will the Devices Be Stored	In a locked location in building 17 where only study staff can access until they are sent out to participants.
Will Devices be supplied at no Cost	No
Is this a HUD (HDE)	No
HDE Number	
Who holds the IDE	N/A
IDE details	

View Details	Device Name
⊟	Home Sleep Test

Manufacturer/Supplier of Device	BetterNight
Where will the Devices Be Stored	In a locked location in building 17 where only study staff can access
Will Devices be supplied at no Cost	No
Is this a HUD (HDE)	No
HDE Number	
Who holds the IDE	N/A
IDE details	

View Details	Device Name
⊟	Ambulatory BP Monitors

Manufacturer/Supplier of Device	VivaLink
Where will the Devices Be Stored	In a locked location in building 17 where only study staff can access

Will Devices be supplied at no Cost	No
Is this a HUD (HDE)	No
HDE Number	
Who holds the IDE	N/A
IDE details	

18.4 Reporting Requirements for FDA-regulated research under IND and IDE:

Describe the process for complying with FDA regulatory requirements for adverse event reporting and adverse device effects reporting to the sponsor

Not Applicable.

18.5 Sponsor (organization/institution/company):

☑ N/A

If applicable, provide sponsor contact information:

19.0 Research Registration Requirements

19.1 ClinicalTrials.gov Registration:

- (●) Registration is not required
- ( ) Registration pending
- ( ) Registration complete

19.2 Defense Technical Information Center Registration (Optional):

- (●) Registration is not required
- ( ) Registration pending
- ( ) Registration complete

20.0 References and Glossary

20.1 References:

1. Stewart IJ, Sosnov JA, Howard JT, et al. Retrospective Analysis of Long-Term Outcomes After Combat Injury. *Circulation*. 2015;132(22):2126-2133. doi:10.1161/CIRCULATIONAHA.115.016950

2. Baker SP, O'Neill B, Haddon W, Long WB. The injury severity score: a method for describing patients with multiple injuries and evaluating emergency care. *J Trauma*. 1974;14(3):187-196. http://www.ncbi.nlm.nih.gov/pubmed/4814394. Accessed May 17, 2017.

3. Stewart IJ, Poltavskiy E, Howard JT, et al. The Enduring Health Consequences of Combat Trauma: a Legacy of Chronic Disease. *J Gen Intern Med*. 2021;36(3):713-721. doi:10.1007/s11606-020-06195-1

4. Duke JM, Randall SM, Wood FM, Boyd JH, Fear MW. Burns and long-term infectious disease morbidity: A population-based study. *Burns*. 2017;43(2): 273-281. doi:10.1016/j.burns.2016.10.020

5. Walker L, Watrous J, Poltavskiy E, et al. Longitudinal mental health outcomes of combat-injured service members. *Brain Behav*. March 2021: e02088. doi:10.1002/brb3.2088

6. American Psychiatric Association. *Diagnostic and Statistical Manual of Mental Disorders: DSM-5*. 5th ed. Washington DC: 2013. doi:10.1176/APPI. BOOKS.9780890425596

7. Haynes ZA, Collen JF, Poltavskiy EA, et al. Risk factors of persistent insomnia among survivors of traumatic injury: a retrospective cohort study. *J Clin Sleep Med*. Published online April 30, 2021. doi:10.5664/JCSM. 92768. J P, PJ M, P L, et al. Baroreflex dysfunction and augmented sympathetic nerve responses during mental stress in veterans with post-traumatic stress disorder. *J Physiol*. 2017;595(14):4893-4908. doi:10.1113 /JP274269

9. O'Donovan A, Ahmadian AJ, Neylan TC, Pacult MA, Edmondson D, Cohen BE. Current posttraumatic stress disorder and exaggerated threat sensitivity associated with elevated inflammation in the Mind Your Heart Study. *Brain Behav Immun*. 2017;60:198-205. doi:10.1016/j.bbi.2016.10.014

10. Bonnet MH, Arand DL. Hyperarousal and insomnia: State of the science. *Slee p Med Rev*. 2010;14(1):9-15. doi:10.1016/j.smrv.2009.05.002

11. Madsen T, Christensen JH, Toft E, Schmidt EB. C-reactive protein is associated with heart rate variability. *Ann Noninvasive Electrocardiol*. 2007; 12(3):216-222. doi:10.1111/j.1542-474X.2007.00164.x

12. Folmer RL, Smith CJ, Boudreau EA, et al. Prevalence and management of sleep disorders in the Veterans Health Administration. *Sleep Med Rev*. 2020; 54:101358. doi:10.1016/j.smrv.2020.101358

13. Moore BA, Tison LM, Palacios JG, Peterson AL, Mysliwiec V. Incidence of insomnia and obstructive sleep apnea in active duty United States military service members. *Sleep*. 2021;44(7). doi:10.1093/sleep/zsab024

14. Caldwell JA, Knapik JJ, Lieberman HR. Trends and factors associated with insomnia and sleep apnea in all United States military service members from 2005 to 2014. *J Sleep Res*. 2017;26(5):665-670. doi:10.1111/jsr.12543

15. Monahan K, Storfer-Isser A, Mehra R, et al. Triggering of Nocturnal Arrhythmias by Sleep-Disordered Breathing Events. *J Am Coll Cardiol*. 2009; 54(19):1797-1804. doi:10.1016/j.jacc.2009.06.038

16. Gami AS, Olson EJ, Shen WK, et al. Obstructive sleep apnea and the risk of sudden cardiac death: A longitudinal study of 10,701 adults. *J Am Coll Cardiol*. 2013;62(7):610-616. doi:10.1016/j.jacc.2013.04.080

17. Lee J-E, Lee CH, Lee SJ, et al. Mortality of Patients with Obstructive Sleep Apnea in Korea. *J Clin Sleep Med*. 2013;9(10):997. doi:10.5664/JCSM.3068

18. Narkiewicz K, Somers VK. The sympathetic nervous system and obstructive sleep apnea: Implications for hypertension. *J Hypertens*. 1997;15(12 Pt 2): 1613-1619. doi:10.1097/00004872-199715120-00062

19. Vgontzas AN, Liao D, Bixler EO, Chrousos GP, Vela-Bueno A. Insomnia with objective short sleep duration is associated with a high risk for hypertension. *Sleep*. 2009;32(4):491-497. doi:10.1093/sleep/32.4.491

20. Gangwisch JE, Malaspina D, Posner K, et al. Insomnia and sleep duration as mediators of the relationship between depression and hypertension incidence. *Am J Hypertens*. 2010;23(1):62-69. doi:10.1038/ajh.2009.202

21. Kibler JL, Joshi K, Ma M. Hypertension in relation to posttraumatic stress disorder and depression in the US National Comorbidity Survey. *Behav Med*. 2009;34(4):125-132. doi:10.3200/BMED.34.4.125-132

22. Player MS, Peterson LE. Anxiety disorders, hypertension, and cardiovascular risk: a review. *Int J Psychiatry Med*. 2011;41(4):365-377. doi:10.2190/PM.41.4.f

23. Howard JT, Sosnov JA, Janak JC, et al. Associations of initial injury severity and posttraumatic stress disorder diagnoses with long-term hypertension risk After combat injury. *Hypertension*. 2018;71(5):824-832. doi:10.1161/HYPERTENSIONAHA.117.10496

24. Kohl HW, Craig CL, Lambert EV, et al. The pandemic of physical inactivity: Global action for public health. *Lancet*. 2012;380(9838):294-305. doi:10.1016/S0140-6736(12)60898-8

25. Rehm J, Gmel G, Sempos CT, Trevisan M. Alcohol-related morbidity and mortality. *Alcohol Res Heal*. 2003;27(1):39-51.

26. Grassi G, Colombo M, Seravalle G, Spaziani D, Mancia G. Dissociation between muscle and skin sympathetic nerve activity in essential hypertension, obesity, and congestive heart failure. *Hypertension*. 1998;31(1):64-67. doi:10.1161/01.HYP.31.1.64

27. Lundin S, Ricksten SE, Thorén P. Renal sympathetic activity in spontaneously hypertensive rats and normotensive controls, as studied by three different methods. *Acta Physiol Scand*. 1984;120(2):265-272. doi:10.1111/j.1748-1716.1984.tb00133.x

28. Hering D, Schlaich M. The role of central nervous system mechanisms in resistant hypertension. *Curr Hypertens Rep*. 2015;17(8). doi:10.1007/s11906-015-0570-0

29. Biasucci LM. C-reactive protein and other markers of inflammation in the prediction of cardiovascular disease in women. *Ital Heart J Suppl*. 2000;1(8):1066-1067. doi:10.1056/nejm200003233421202

30. Ridker PM, Cushman M, Stampfer MJ, Tracy RP, Hennekens CH. Inflammation, aspirin, and the risk of cardiovascular disease in apparently healthy men. *NEJM*. 1997;336(14):973-979. doi:10.1056/NEJM199704033361401

31. Walker LE, McCabe CT, Watrous JR, et al. Prospective evaluation of health outcomes in a nationwide sample of aeromedical evacuation casualties: methods from a pilot study. *Mil Med*. Publisehd online April 4, 2021. doi:10.1093/milmed/usab329

32. Whelton PK, Carey RM, Aronow WS, et al. 2017 ACC/AHA/AAPA/ABC/ACPM/AGS/APhA/ASH/ASPC/NMA/PCNA Guideline for the Prevention, Detection, Evaluation, and Management of High Blood Pressure in Adults: Executive Summary: A Report of the American College of Cardiology/American Heart Association Task Force on Clinical Practice Guidelines. *Hypertension*. 2018;71(6):1269-1324. doi:10.1161/HYP.0000000000000066

33. Staessen JA, Yang WY, Melgarejo JD, et al. Association of office and ambulatory blood pressure with mortality and cardiovascular outcomes. *JAMA*. 2019;322(5):409-420. doi:10.1001/jama.2019.9811

34. Dobson CP, La Rovere MT, Pinna GD, et al. QT variability index on 24-hour Holter independently predicts mortality in patients with heart failure: Analysis of Gruppo Italiano per lo Studio della Sopravvivenza nell' Insufficienza Cardiaca (GISSI-HF) trial. *Hear Rhythm*. 2011;8(8):1237-1242. doi:10.1016/j.hrthm.2011.03.055

35. Berger RD, Kasper EK, Baughman KL, Marban E, Calkins H, Tomaselli GF. Beat-to-beat QT interval variability: Novel evidence for repolarization lability in ischemic and nonischemic dilated cardiomyopathy. *Circulation*. 1997;96(5):1557-1565. doi:10.1161/01.CIR.96.5.1557

36. Haigney MC, Kop WJ, Alam S, et al. QT variability during rest and exercise in patients with implantable cardioverter defibrillators and healthy controls. *Ann Noninvasive Electrocardiol*. 2009;14(1):40-49. doi:10.1111/j.1542-474X.2008.00274.x

37. Haigney MCP, Alam S, Tebo S, et al. Intravenous cocaine and QT variability. *J Cardiovasc Electrophysiol*. 2006;17(6):610-616. doi:10.1111/j.1540-8167.2006.00421.x

38. Haigney MC, Zareba W, Nasir JM, et al. Gender differences and risk of ventricular tachycardia or ventricular fibrillation. *Hear Rhythm*. 2009;6(2):180-186. doi:10.1016/j.hrthm.2008.10.045

39. Solhjoo S, Punjabi NM, Ivanescu AE, et al. Methadone destabilizes cardiac repolarization during sleep. *Clin Pharmacol Ther*. Published online July 19, 2021. doi:10.1002/cpt.2368

40. Dobson CP, La Rovere MT, Olsen C, et al. 24-Hour QT variability in heart failure. *J Electrocardiol*. 2009;42(6):500-504. doi:10.1016/j.jelectrocard.2009.06.021

41. Haigney MC, Zareba W, Gentlesk PJ, et al. QT interval variability and spontaneous ventricular tachycardia or fibrillation in the Multicenter Automatic Defibrillator Implantation Trial (MADUT) II patients. *J Am Coll Cardiol*. 2004;44(7):1481-1487. doi:10.1016/j.jacc.2004.06.063

42. Weathers FW, Litz BT, Keane TM, Palmieri PA, Marx BP, Schnurr PP. The PTSD Checklist for DSM-5 (PCL-5). Scale available from the National Center for PTSD; 2013. www.ptsd.va.gov.

43. Kroenke K, Strine TW, Spitzer RL, Williams JBW, Berry JT, Mokdad AH. The PHQ-8 as a measure of current depression in the general population. *J Affect Disord*. 2009;114(1-3):163-173. doi:10.1016/j.jad.2008.06.026

20.2 Abbreviations and Acronyms:

hypertension (HTN)
cardiovascular disease (CVD)
post-traumatic stress disorder (PTSD)
intensive care unit (ICU)
Department of Defense (DoD)
Injury Severity Score (ISS)
hazard ratio (HR)
sympathetic nervous system (SNS)
obstructive sleep apnea (OSA)
Wounded Warrior Recovery Project (WWRP)
electrocardiogram (ECG)

heart rate variability (HRV)<br>QT interval and its variability (QTV)<br>premature ventricular contractions (PVC)<br>home sleep apnea testing (HST)<br>ambulatory blood pressure monitor (ABPM)<br>continuous positive airway pressure (CPAP)<br>PTSD checklist for DSM-5 (PCL-5)<br>Brief dissociative experience scale (DES-B)<br>Patient Health Questionnaire 8 (PHQ-8)<br>Pittsburgh Sleep Quality Index (PSQI)<br>Epworth Sleepiness Scale (ESS)<br>Insomnia Severity Index (ISI)	
